# Inhibition of calpain-mediated BAX cleavage attenuates Japanese encephalitis virus-induced apoptosis in SH-SY5Y neuroblastoma cells

**DOI:** 10.1186/s12985-026-03242-x

**Published:** 2026-07-02

**Authors:** Kuntida Kitidee, Arisara Samutpong, Nattaporn Pakpian, Montri Yasawong, Prapimpun Wongchitrat

**Affiliations:** 1 Center for Research Innovation and Biomedical Informatics, Faculty of Medical Technology, Mahidol University, 999 Phutthamonthon 4 Road, Salaya, Nakon Pathom, 73170 Thailand; 2 Department of Pathology, Faculty of Medicine, Ramathibodi Hospital, Mahidol University, Bangkok, Thailand; 3 Faculty of Medicine Ramathibodi Hospital, Chakri Naruebodindra Medical Institute, Mahidol University, Samut Prakan, Thailand; 4 Program on Environmental Toxicology, Chulabhorn Graduate Institute, Bangkok, Thailand

**Keywords:** Japanese encephalitis virus, Calpain, Calpeptin, p18 BAX, Apoptosis

## Abstract

**Background:**

Japanese encephalitis virus (JEV) is an arbovirus which is the most common virus in the genus *Orthoflavivirus* that causes significant viral encephalitis with a high fatality rate and is distributed throughout Asian countries. JEV infection induces brain tissue disease characterized by neuronal death. We previously demonstrated that JEV elevates the formation of an N-terminal truncated version of BAX (p18 BAX), triggering mitochondria-mediated apoptosis. However, the mechanisms by which virus-host interactions induce the generation of p18 BAX during JEV infection are unclear.

**Methods:**

The present study aims to investigate the underlying mechanism by which JEV-induced cell apoptosis is mediated by the activation of calpain-induced proteolytic cleavage of BAX. Calpeptin, a calpain inhibitor, was used to evaluate the effect on calpain activity, cell viability, and apoptotic molecules expression in JEV-infected neuroblastoma SH-SY5Y cells.

**Results:**

Our data showed that JEV infection resulted in the activation of the calpain-mediated apoptosis pathway, as evidenced by increased calpain activation and p18 BAX generation. The translocation of p18 BAX to the mitochondria results in cytochrome c release and increased of caspase-3 activity, resulting in an increase of cleaved PARP expression, which induced neuronal apoptosis. Inhibiting calpain activity by calpeptin attenuated all these effects.

**Conclusions:**

Our results reveal that JEV induces neuronal cell apoptosis by activating calpain-mediated cleavage of BAX. These findings provide a novel insight into JEV-caused encephalitis and suggest that calpain inhibition may represent a potential therapeutic strategy.

**Supplementary Information:**

The online version contains supplementary material available at https://doi.org/10.1186/s12985-026-03242-x.

## Background

Japanese encephalitis virus (JEV) is an arbovirus that causes Japanese encephalitis, a severe neurological disease with high mortality and morbidity. Japanese encephalitis affects almost 60% of the global population lives which is endemic in most of Southeast Asia, along with northern Australia and some parts of the Western Pacific [1]. Most of the patients infected with JEV are subclinical only 1:1000 develop severe neurological symptoms. Approximately 20–30% of clinical Japanese encephalitis cases are fatal and a high proportion of survivors suffer from serious neurologic, cognitive, or psychiatric complications even years later that burden the daily life of both the patients and caretakers [2, 3]. No specific drugs are available to treat Japanese encephalitis only supportive treatment is managed to lower the death rate. Due to its zoonotic cycle of transmission and ineffective mosquito control, vaccination is currently the only available control measure [4].

JEV is a positive-sense single-stranded virus belonging to the family *Flaviviridae*, which includes dengue, West Nile, and Zika viruses. Japanese encephalitis virus is an enveloped, spherical particle ~ 50 nm in diameter. The viral genome consists of genes encoding three structural proteins, including capsid (C), membrane (prM), and envelope (E), and seven nonstructural proteins (NS1, NS2A, NS2B, NS3, NS4A, NS4B, and NS5) [5, 6]. JEV mainly affects the central nervous system (CNS) by crossing the blood-brain barrier (BBB) and entering various types of neuronal cells [7]. The JEV infection induces brain tissue disease characterized by neuron death. Several reports show that neuronal apoptosis is an important process attributed to JEV pathogenesis. The infection of JEV causes the elevation of oxidants, which induces cellular stress and leads to the activation of the apoptosis pathway [2, 4]. The induction of apoptosis molecules includes pro-apoptotic and anti-apoptotic in B cell lymphoma-2 (BCL-2) family proteins members play a crucial role in JEV-induced neuronal death [8, 9]. A slow progression of the JEV-induced apoptotic cell death is presented at the early stage of infection, whereas the strong activation of pro-apoptotic molecules to trigger cell apoptosis happened during the late period of infection [10, 11]. The JEV infection induces an increase in the expression and translocation of apoptosis regulator Bcl-2-associated X protein (BAX) from the cytosol to mitochondria to promote the release of cytochrome c (Cyt *c*) and induce caspase cascade activation lead neuronal cells undergo apoptotic cell death [10, 12, 13]. In addition to the pro-apoptotic effect of BAX translocation, our previous study demonstrates the proteolytic cleavage of BAX at the N-terminus generates a potent pro-apoptotic 18 kDa fragment (p18 BAX) that appears to enhance apoptotic cell death, especially at the late period of JEV infection [10]. The p18 BAX fragment is considered a potent pro-apoptotic molecule with high toxicity to the cells. As evidenced in several of the studies demonstrate that active calpains have been shown to cleave a full length of BAX (p21) at its N-terminus (at Asp33), generating a truncated 18-kDa fragment and thus enabling it to homodimerize and enter mitochondria [14]. However, the underlying molecular mechanisms of JEV-induced p18 BAX accumulation in the neurons remain unclear.

Calpains are a family of calcium-dependent neutral cysteine proteases for which two major isoforms, calpain 1 and calpain 2, are ubiquitously expressed and control the proteolysis of a broad spectrum of substrates upon activation [15]. Calpains are implicated in various physiological and pathological processes, including cell proliferation, apoptosis, autophagy, and inflammation [16–18]. It has been demonstrated that virus infections can induce host cell death through the activation of the calpain mechanism. The alteration of calpain activity is reported to play a role in apoptosis during the pathogenic process associated with viral infections [19–23]. The inhibition of calpain showed to ameliorate the severity of viral-induce host cell damage and suppress the production of new viral progeny both in vivo and in vitro of several viruses such as influenza A virus (IAV) [24], enterovirus 71 (EV71) [22, 23], Chikungunya virus (CHIKV) [25], Coxsackievirus B3 (CBV3) [26] and SARS-CoV-2 [27, 28]. However, whether calpain activation is associated with the apoptotic pathway in JEV-infected cells has yet to be determined.

In the present study, we investigate the underlying mechanism by which JEV-induced apoptosis is mediated through the activation of calpain-induced proteolytic cleavage of BAX, and whether calpain inhibition could reduce neuronal apoptotic cell death during JEV infection.

## Materials and methods

### Chemicals and reagents

Calpeptin (ab120804) was purchased from Abcam (Cambridge, UK). Dimethyl sulfoxide (DMSO) was obtained from Calbiochem^®^ by Millipore (Bedford, MA, USA). Dulbecco’s Modified Eagle’s medium (DMEM), fetal bovine serum (FBS), and amphotericin B were purchased from Gibco (NY, USA). Mouse monoclonal anti-BCL-2, rabbit monoclonal anti-BAX, rabbit polyclonal anti-poly (ADP-ribose) polymerase (PARP), horseradish peroxidase (HRP)-conjugated anti-rabbit IgG and anti-mouse IgG antibodies were purchased from Cell Signaling Tech, Inc. (Danvers, MA, USA). Mouse monoclonal anti-spectrin alpha chain (alpha II-spectrin), rabbit polyclonal anti-caspase 3, and mouse monoclonal anti-actin were obtained from Millipore (Madison, WI, USA). CellTiter 96^®^ AQueous One Solution Cell Proliferation for [3-(4,5-dimethylthiazol-2-yl)−5-(3-carboxymethoxyphenyl)−2-(4-sulfophenyl)−2 H-tetrazolium] (MTS) assay were purchased from Promega (Madison, WI, USA). Protein Assay Dye Reagent Concentrate for Bradford assay was purchased from Bio-Rad Laboratories (Hercules, CA, USA). Luminata™ Forte Western HRO substrate was purchased from Millipore.

### Cell lines

The SH-SY5Y human neuroblastoma cells (ATCC No.CRL-2266) and the Vero epithelial cell line (ATCC No. CCL-81) were used in this study. SH-SY5Y and Vero cells were cultured in 75-cm^2^ flasks in DMEM supplemented with 10% heat-inactivated FBS, amphotericin B, and antibiotics. Cells were incubated in a 5% CO_2_ humidified incubator at 37 °C. Both cell lines were maintained and subcultured to obtain a sufficient cell number for the subsequent experiment.

### Virus

The JEV strain Beijing-1 was kindly provided by Prof. Dr. Sutee Yoksan at the Center for Vaccine Development, Institute of Molecular Biosciences, Mahidol University. JEV strain Beijing-1 was confirmed by sequence analysis using specific JEV E gene primers (GenBank accession No. L48961). The virus was propagated in Vero cells by inoculating with JEV at a multiplicity of infection (MOI) of 0.01 in 2% FBS DMEM at 37 °C and 5% CO₂ for 2 h with occasional shaking. After removing the inoculum and washing with 2% FBS DMEM, infected cells were cultured in 2% FBS EMEM until cytopathic effects (CPE) reached 3–4 + degrees. Culture supernatants were collected, centrifuged at 2,000 rpm for 20 min at 4 °C, aliquoted, and stored at −80 °C. Viral titers were determined by plaque assay as previously described [10, 11].

### Cytotoxicity of calpeptin

Calpeptin, a cell-permeable calpain inhibitor, was freshly prepared by dissolved in DMSO to 10 mM solution and then further diluted in a culture medium with 2% FBS to obtain concentrations of 1, 5, 10, and 50 µM. For treatment, SH-SY5Y cells were seeded in 96-well plates at a density of 6 × 10^4^ cells per well until they reached 80–90% confluence in 24 h and treated with various concentrations of calpeptin for the final 24 h prior to harvesting at 48 h. Assays were run in quadruplicates, and a 2% FBS-supplemented medium without calpeptin (0 µM) was used as a control. Cell viability was evaluated by MTS assays.

### Calpain inhibitor treatment

A confluent monolayer of SH-SY5Y cells was prepared in microplates and pretreated with 10 µM calpeptin for 1 h. Calpeptin was then removed, and cells were infected with JEV at an MOI of 1 for 1 h. Then, the excess virus was removed by washing with a culture medium. Infected cells were further incubated in a standard culture medium for 24 h and then treated with the 10 µM calpeptin for an additional 24 h before the next analysis or harvesting of the cells. The CPE was observed and photographed under a light microscope. A schematic of the virus infection and the treatment procedure is detailed in Fig. 2A.

### Detection of cell viability by MTS assay

SH-SY5Y cells were seeded in 96-well plates at a density of 6 × 10^4^ cells per well for 24 h and then treated as described above. After treatment, cells were investigated for viability by MTS assay using CellTiter 96^®^ AQueous One Solution Cell Proliferation according to the manufacturer’s protocol. Briefly, 20 µL assay reagent was added to each well containing treated cells in 100 µL of culture medium and incubated at 37 °C and 5% CO_2_ for 3 h. The optical density at 490 nm was measured by Thermo Scientific™ Multiskan SkyHigh Microplate Spectrophotometer (Thermo Fisher Scientific Inc., CA, USA). The percentage of cell viability was calculated relative to untreated control cells, designated as 100%.

### Western blot analysis

In another set of experiments, in order to investigate the effect of calpain inhibitor, calpeptin, treatment on the induction of calpain-mediated neuronal cell death by JEV infection, the expression of apoptosis-associated proteins was investigated by using western blot analysis. SH-SY5Y cells were seeded in 24-well plates at a density of 3 × 10^5^ cells per well for 24 h and then treated as described above. After treatment, the treated JEV-infected SH-SY5Y cells were collected at 48 h after infection. The cells were lysed in RIPA buffer supplement with 1% protease inhibitor cocktail and centrifuged at 12,000 g for 15 min at 4 °C, then collected supernatant, and the total protein concentration was measured using a Bradford assay. Samples were separated by SDS-PAGE gel electrophoresis and transferred onto a polyvinylidene difluoride membrane. Membranes were blocked with 5% skim milk and then probed overnight at 4 °C with antibodies against BCL-2 (1:1,000), BAX (1:1,000), Caspase-3 (1:400), PARP (1:1,000), and Actin (1:10,000). After washing with TBST, membranes were incubated with the corresponding HRP-conjugated secondary antibody for 1.5 h at room temperature. Immunoblots were visualized using the Luminata™ Forte Western HRO substrate, and captured chemiluminescent signals by ChemiDoc™ MP imager (Bio-Rad Laboratories). Actin was used as a loading control for normalization. Band densities were measured using ImageJ software (NIH, Bethesda, MD, USA). Each target protein band was first normalized to the corresponding actin band. The resulting normalized density for each experimental group was then expressed as fold change relative to the normalized density of the uninfected control group.

### Calpain and caspase-3 activities

Calpain and caspase-3 activities in JEV-infected SH-SY5Y cells with or without calpeptin treatment were assessed by western blot analysis using an anti-αII-spectrin antibody (1:1,000). αII-Spectrin (~ 280 kDa) serves as a dual-reporter substrate: calpain-1 and calpain-2 cleave it to produce a 145 kDa spectrin breakdown product (SBDP), whereas caspase-3 cleaves the same substrate at a distinct site to generate a 120 kDa SBDP [24, 29, 30]. Detection of both fragments from a single blot therefore allows simultaneous and independent assessment of calpain and caspase-3 activities, with increases in the 145 kDa and 120 kDa SBDPs serving as indicators of calpain and caspase-3 activation, respectively.

### Determination of mitochondrial BAX and cytochrome *c*

In order to ensure that JEV infection induces calpain-mediated BAX aggregation in mitochondria, which then activates the intrinsic apoptotic pathway. The effect of calpeptin on the mitochondrial p18 BAX and Cyt *c* expression was determined. The mitochondrial fractionations were prepared using the isolation kit (Abcam, Cambridge, UK) according to the instruction of the manufacturer. Briefly, approximately 1.2 × 10^6^ SH-SY5Y cells were prepared and then infected with JEV at an MOI of 1 and treated with calpeptin as described above then the treated cells were harvested at 48 hpi. The cells were gently pelleted by low speed centrifugation (600 g), washed once in ice-cold PBS, pelleted again and re-suspend cells with 1X cytosolic buffer mix containing protease inhibitors and DTT and incubated on ice for 10 min. Cells were subjected for homogenization with syringe 27G gauge. The efficiency of homogenization was assessed by microscopy when 70–80% of the cells lacked the shiny ring. Then the homogenates were transferred to a 1.5 ml microcentrifuge tube and centrifuged (700 g) for 10 min at 4 °C. The supernatants were transferred to a fresh microcentrifuge tube and centrifuged at 10,000 g for 30 min at 4 °C. The pellets were re-suspended with the mitochondrial buffer mix containing protease inhibitors and DTT and mixed by vortex for 10 s, then this solution was designated as mitochondrial fractions. The translocation of p18 BAX to mitochondria and the presence of Cyt *c* was determined by western blotting, using a rabbit monoclonal anti-human BAX (1:1000) and a rabbit monoclonal anti-human Cyt *c* antibody (1:500, Cell Signaling Technology, Inc.). The fraction purity was confirmed using anti-cytochrome oxidase IV (Cox IV; 1:2,500; Cell Signaling Technology, Inc.) as a mitochondrial marker and anti-actin (1:10,000; MilliporeSigma) as a cytosolic marker.

### Digital PCR

To investigate the effect of calpeptin treatment on JEV replication, absolute quantification of JEV genomic RNA from culture supernatants at 48 hpi was performed using digital PCR (dPCR). Viral RNA was extracted from culture supernatants using the magLEAD^®^ 12gC automation system using MagDEA^®^ Dx Nucleic Acid Extractions reagent (Precision System Science Co., Ltd., Japan) according to the manufacturer’s instructions. Culture supernatant from JEV-infected cells without calpeptin treatment was used as the JEV control. The RNA was aliquoted and stored at −80 °C until used for quantifying JEV viral load by dPCR.

Reverse transcriptase real-time PCR (RT-qPCR) was performed during method optimization for JEV NS1, NS3, and NS5 primers and probes concentrations and annealing temperatures. RT-qPCR was performed using SuperScript III Platinum One-Step qRT-PCR System (Invitrogen, Thermo Fisher Scientific Inc.). Primer and probe sequences for JEV genes were as follows: NS1; F 5’-GCCACCCAGGAGGTCCTT-3’, R 5’-CCCCAAAACCGCAGGAAT-3’ and probe 5’FAM-CAAGAGGTGGACGGCC-BHQ13’, NS3; F 5’-AGAGCACCAAGGGAATGAAATAGT-3’, R 5’-AATAGGTTATAGTTGGGCACTCTGT-3’ and probe 5’FAM-CCACGCCACTCTGACCCACAGACTG-BHQ1 3’, and NS5; F 5’-TGGACCACAGCACTTGGAACA-3’, R 5’-CACAGTCATCTCCGCTGATC-3’ and probe 5’FAM-CTTTGAGAATGGAGAGGAGAGAGTGA-BHQ13’ All primers and probes were synthesized by Integrated DNA Technologies (Singapore). The no-template control was not detected by RT-qPCR, confirming no contamination of the experiment performing and extraction processes.

Using the optimized conditions, reverse transcriptase digital PCR (RT-dPCR) was performed using a QIAcuity Digital PCR System (QIAGEN, Hilden, Belgium) for absolute quantification of JEV NS1, NS3, and NS5 concentrations. RNA extracts were assayed with the QIAcuity^®^ One-Step Viral RT-PCR Kit (catalog no.1123145, QIAGEN) and 8,500 24-well Nanoplates (catalog no. 250011, QIAGEN) according to the manufacturer’s recommendations. Each RT-dPCR reaction contained 3.6 µL of master mix, 400 nM forward primer, 400 nM reverse primer, 200 nM probe, 0.12 µL of 100× Multiplex Reverse Transcription Mix, 3.28 µL of nuclease-free water, and 5 µL of template RNA. The amplification steps using the thermal cycling protocol were as follows: 50 °C for 40 min for reverse transcription, 95 °C for 2 min for enzyme activation, 95 °C for 5 s for denaturation, and 60 °C for 30 s for annealing/extension for 45 cycles. Images were acquired using an exposure time of 300 ms in the FAM channel. No template control and negative control were included in each run to set the fluorescence threshold for the probe-primer set. All samples were tested in duplicate, and results were analyzed by the QIAcuity Suite Software (QIAGEN).

### Plaque assay

To investigate the effect of calpeptin treatment on JEV replication, viral titers in experimental culture supernatants were determined by plaque assay at 48 hpi. Vero cells were seeded in 12-well culture plates and, after 24 h, cell monolayers were infected with serial 10-fold dilutions of supernatants in triplicate. Inoculated cells were incubated at 37 °C and 5% CO₂ for 2 h with intermittent shaking for virus adsorption. The monolayers were overlaid with 1.6% carboxymethylcellulose in 2% FBS EMEM and incubated at 37 °C and 5% CO_2_ for 6 days. Cells were stained with crystal violet after fixation with 10% formaldehyde at room temperature for 2 h. Plaques were counted and titers calculated as plaque-forming units (pfu/mL). Results are presented as the mean of triplicate wells.

### Statistical analysis

Statistical analyses were performed with GraphPad Prism software (GraphPad, San Diego, CA, USA). Data are presented as mean ± standard deviation (SD). Data are presented as mean ± SD from at least three independent experiments. Differences between groups were analyzed using one-way analysis of variance (ANOVA) followed by a comparison test as appropriate. A p-value < 0.05 was considered statistically significant.

## Results

### Cytotoxicity of calpeptin on SH-SY5Y cells

Based on our previously published kinetic studies of JEV infection in SH-SY5Y cells [10, 11], significant neuronal apoptosis was first detected at 48 hpi in cells infected at an MOI of 1. At the earlier time point of 24 hpi, cytopathic effects were absent or minimal, no significant differences in apoptotic rate were observed, and p18 BAX levels were undetectable or negligible compared to uninfected control cells. Accordingly, 48 hpi represents the time point at which calpain-mediated effects which including p18 BAX accumulation and its downstream apoptotic consequences that are most pronounced and reliably detectable. All subsequent experiments in this study were therefore performed at 48 hpi.

**Fig. 1:**
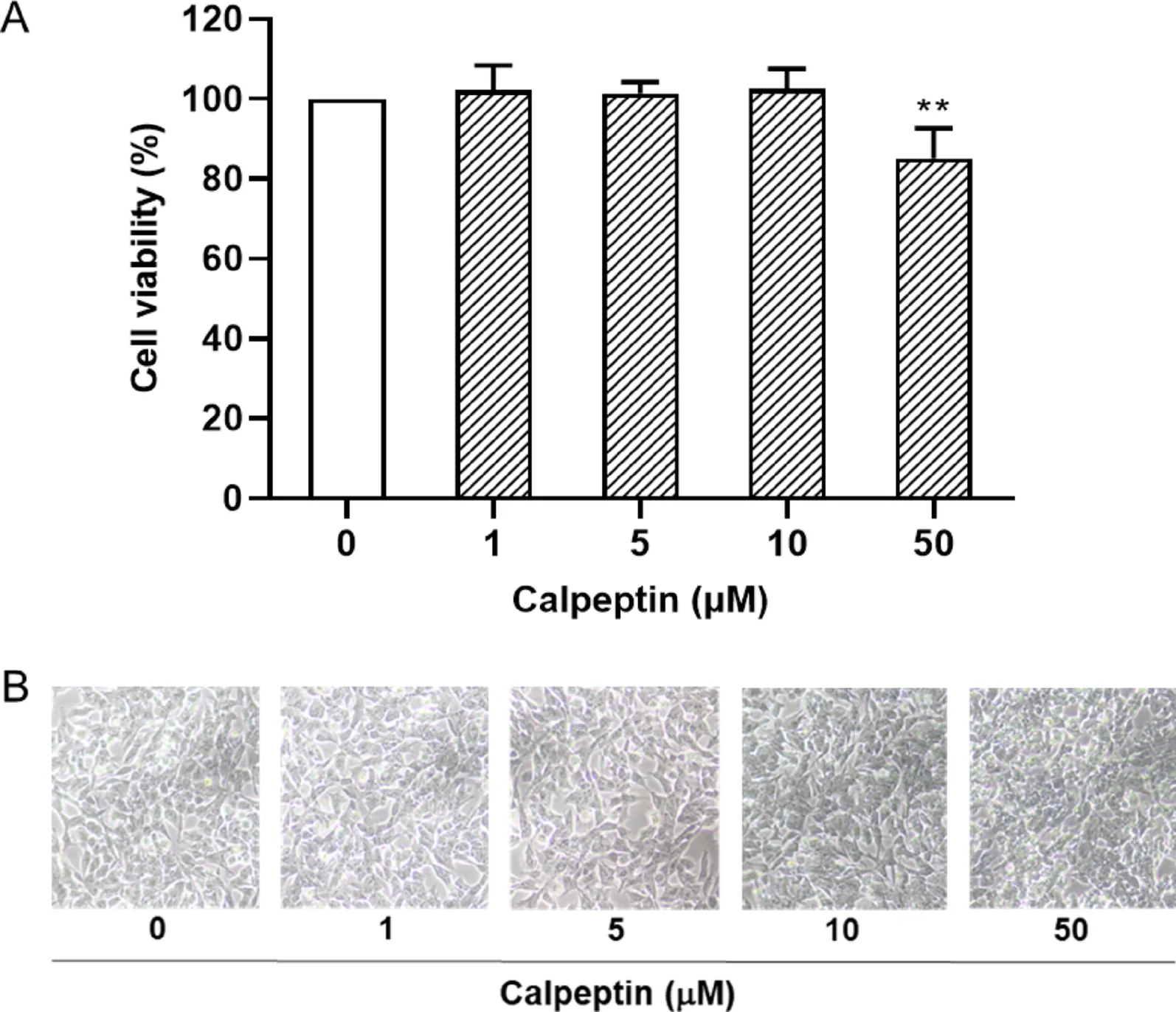
Cytotoxicity test of calpeptin. (**A**) Cell viability of SH-SY5Y cells at 48 h. Calpeptin was serially diluted to concentrations of 1, 5, 10, and 50 µM. SH-SY5Y cells were treated with various calpeptin for the final 24 h prior to harvesting the cells at 48 h. Subsequently, the cytotoxicity of calpeptin was determined by MTS assay. The optical density of the reaction was measured at a wavelength of 490 nm. The percentage of cell viability is presented compared to the cell control (0 µM calpeptin). The results are indicated as the means ± SD of four independent experiments. One-way ANOVA and the Tukey-Kramer multiple comparisons test were performed for statistical analysis. **p < 0.01 compared with control. (**B**) Cell morphology of SH-SY5Y cells observed under a light microscope at 48 h (magnification, 10 × 20). The representative image for morphological change of cells after being treated with calpeptin at different concentrations is shown

As demonstrated in our previous studies, JEV infection significantly induces apoptosis in SH-SY5Y cells, particularly at the late stage of infection [10, 11]. Accordingly, all experiments in this study were performed at 48 h. We first evaluated the cytotoxicity of calpeptin, an inhibitor of calpain 1 and calpain 2, on SH-SY5Y cells at 48 h post-treatment using the MTS assay. Calpeptin at 1, 5, 10, and 50 µM concentrations was treated to the SH-SY5Y cells for 48 h. No cytotoxic effect at 1–10 µM by either MTS assay (Fig. 1A) or morphological observation (Fig. 1B), except at 50 µM, which significantly reduced cell viability compared to the untreated (0 µM) control. As corresponding to previous reports, the nontoxic dose of calpeptin at 10 µM, which exhibited a percentage of cell viability of more than 95%, was selected for further experiments.

### Inhibition of calpain prevents JEV-induced cell death

**Fig. 2:**
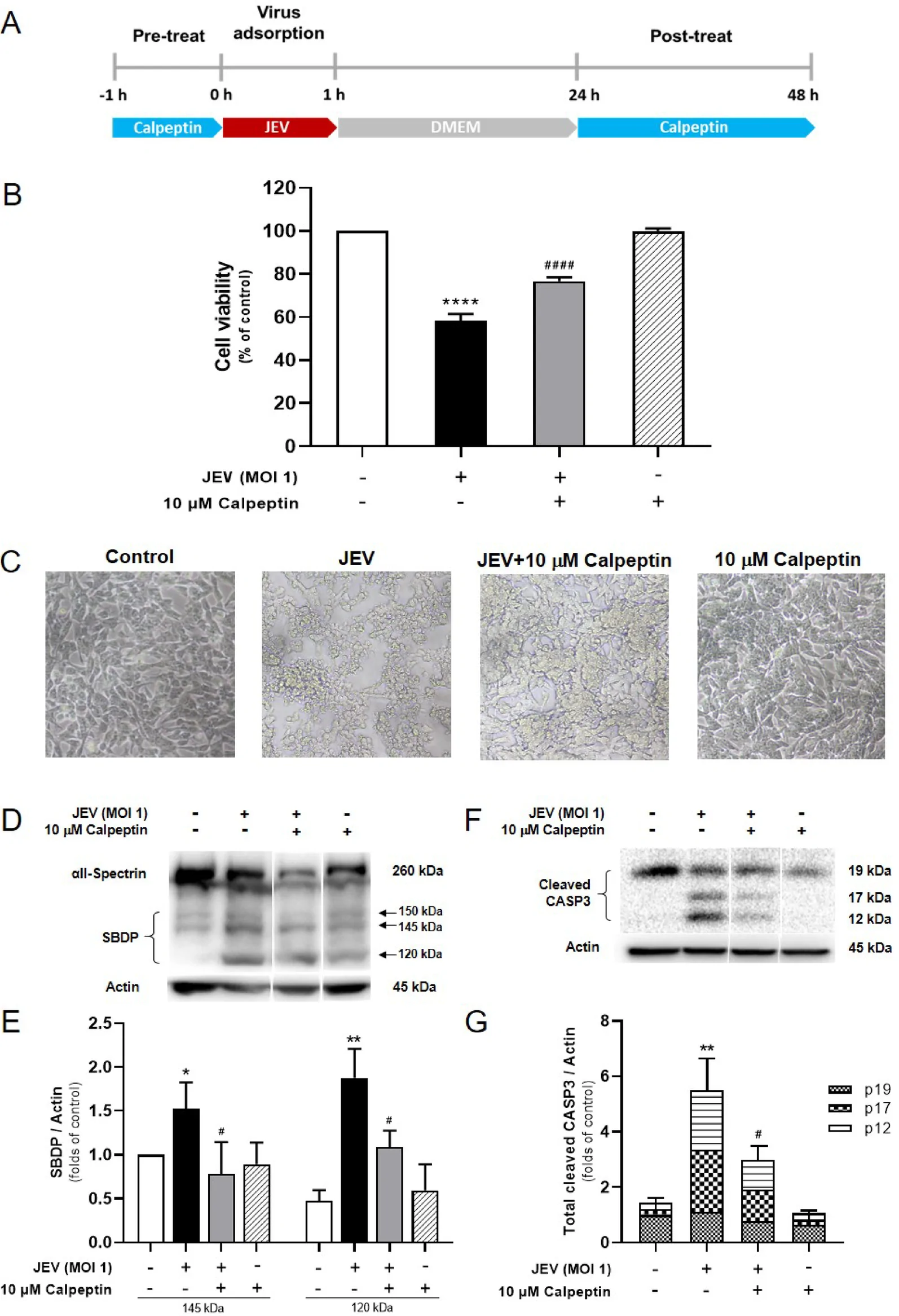
The effect of JEV infection on cell viability, calpain, and caspase-3 activities in SH-SY5Y, and the effect of calpeptin treatment at 48 h. (**A**) Schematic representation of the experimental design. The red bar indicates the JEV adsorption period; the blue bar indicates the period of calpeptin treatment. SH-SY5Y cells were pretreated with 10 µM calpeptin for 1 h prior to JEV infection at MOI 1, maintained in standard medium for 24 h, and then treated with 10 µM calpeptin for a further 24 h before harvesting at 48 hpi. (**B**) Cell viability measured by MTS assay. Results represent means ± SD from three independent experiments. ****p < 0.0001 compared to the cell control. ^####^*p* < 0.0001 compared with the JEV-infected cells. (**C**) Cytopathic effect (CPE) in JEV-infected SH-SY5Y cells (magnification, 10 × 20). **D**-**E**) Western blot detection and densitometric quantification of αII-spectrin breakdown products (SBDPs); 145-kDa SBDP (generated by calpain-specific cleavage of αII-spectrin) and 120-kDa SBDP (generated by caspase-3-specific cleavage of αII-spectrin). **F**-**G**) Western blot detection and densitometric quantification of cleaved caspase-3 subunits (p19, p17, and p12). The immunoblot shown is from one representative experiment. Densitometric analysis values represent means ± SD of three independent experiments. One-way ANOVA and Tukey–Kramer multiple comparison tests were performed for statistical analysis. *p < 0.05 and **p < 0.01 compared to the cell control. #*p* < 0.05 compared with the JEV-infected cells. Immunoreactive bands from different regions of the same blot were extracted as indicated by the dividing line. The original blots are presented in Supplementary Figs. 1 and 2

To test the hypothesis that JEV induces neuronal death through the calpain-mediated pathway, a calpeptin was used to evaluate the effect on cell viability in JEV-infected SH-SY5Y cells. Calpeptin at a concentration of 10 µM was treated in the JEV-infected cells for 48 h. The results showed that JEV infection of SH-SY5Y cells at an MOI of 1 caused a significant decrease in cell viability to 58.74% ± 2.86 as compared to uninfected control cells (*p* < 0.001, Fig. 2B). JEV-induced cytopathic effects in SH-SY5Y cells confirmed the viral invasion, which corresponded to the decrease in cell viability results (Fig. 2C). Treatment with 10 µM calpeptin was shown to reduce the induction of cell death by JEV infection. In comparison with the untreated JEV-infected groups, the cell viability exhibited a significant increase to 76.26% ± 1.76 in the 10 µM calpeptin-treated JEV-infected groups (*p* < 0.001, Fig. 2B). These results demonstrate that suppression of calpain activity by calpeptin attenuates JEV-induced neuronal death.

### JEV infection activates calpain activity and upregulates caspase-3 activity in SH-SY5Y cells

To investigate the activation of calpain activity during JEV infection in neuronal cells, we performed western blotting to assess the calpain activity by the formation of calpain-specific 145 kDa SBDP at 48 h post-infection (hpi). As shown in Fig. 2D, the 145 kDa SBDP was elevated in JEV-infected cells compared to uninfected control cells (*p* < 0.05; Fig. 2E), indicating increased calpain activity. Concurrently, the 120 kDa SBDP was also significantly increased in JEV-infected cells (*p* < 0.01; Fig. 2E), reflecting caspase-3 activation. Moreover, the caspase-3 activity was further confirmed by the formation of cleaved caspase-3 subunits in the infected cells. Western blot analysis showed that the p19 intermediate product was detected in uninfected control cells, while JEV-infected cells at 48 hpi displayed the full set of p19, p17, and p12 subunits (Fig. 2F), with p17 and p12 representing fully processed, enzymatically active caspase-3. The total level of cleaved caspase-3 fragments was elevated in JEV-infected cells compared to controls (*p* < 0.05; Fig. 2G). The increase of calpain and caspase-3 activities by JEV corresponded well with the increase in cell death in the JEV-infected group (Fig. 2B and C). These findings indicate that JEV infection induces neuronal death through coordinated activation of the calpain- and caspase-3-mediated apoptosis pathway.

### Inhibition of calpain activity by calpeptin reduces caspase-3 activity during JEV infection

We next examined the effect of calpeptin treatment on the calpain and caspase-3 activities in JEV-infected cells. Calpeptin treatment significantly reduced the elevation in calpain activity induced by JEV infection, as evidenced by a decrease in the 145 kDa SBDP in calpeptin-treated infected cells compared to untreated infected cells (*p* < 0.05; Fig. 2D and E). Correspondingly, calpeptin treatment also reduced the caspase-3-dependent 120 kDa SBDP (Fig. 2D and E) and the level of cleaved caspase-3 fragments (*p* < 0.05; Fig. 2G) which is a typical hallmark of apoptosis [31]. The reduction in both calpain and caspase-3 activities in calpeptin-treated infected cells was accompanied by improved cell viability (Fig. 2B). Treatment with 10 µM of calpeptin alone had no effect on either calpain or caspase-3 activity in SH-SY5Y cells. These findings suggest that the JEV infection induces neuronal apoptosis through a calpain- and caspase-3-mediated pathway. The inhibition of calpain activity has been demonstrated to enhance cell survival.

### Inhibition of calpain activity suppresses JEV-induced generation of p18 BAX in SH-SY5Y

**Fig. 3:**
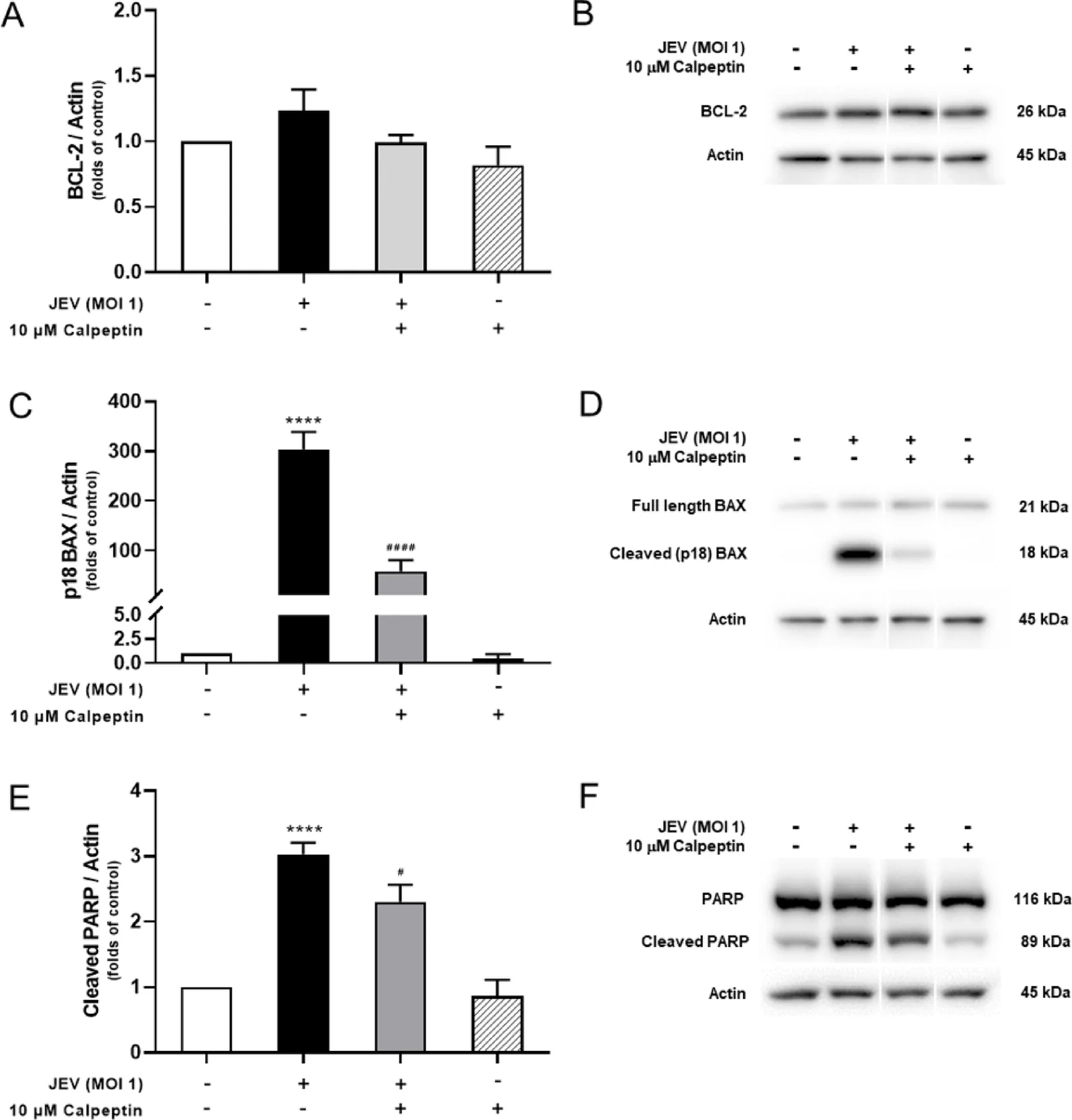
The effect of calpeptin on the apoptosis signaling pathway in JEV-infected SH-SY5Y cells. SH-SY5Y cells were pretreated with 10 µM calpeptin for 1 h prior to JEV infection at MOI 1, maintained in standard medium for 24 h, and then treated with 10 µM calpeptin for a further 24 h before harvesting at 48 hpi. The infected cells were collected, and the total protein was extracted at 48 h. The immunoreactive detection and respective quantification of **A**-**B**) BCL-2, **C**-**D**) p18 BAX, and **E**-**F**) PARP were analyzed by western blotting. The immunoblots shown are from one representative experiment. Densitometric analysis values represent means ± SD of three independent experiments. One-way ANOVA and Tukey–Kramer multiple comparison tests were performed for statistical analysis. ****p < 0.0001 compared to the cell control. #*p* < 0.05, and ^####^*p* < 0.0001 compared with the JEV-infected cells. Immunoreactive bands from different regions of the same blot were extracted as indicated by dividing line. The original blots are presented in Supplementary Fig. 3 to 7

As previously demonstrated, JEV infection remarkably elevates the expression levels of the p18 BAX fragment in SH-SY5Y cells, contributing to neuronal apoptosis at the late stage of infection [10]. In this study, we sought to determine whether calpain-mediated cleavage of BAX occurs during JEV infection. Western blot analysis was performed to evaluate the presence of cleaved BAX (p18) and full-length BAX (p21), as well as BCL-2, in infected cells treated with or without calpeptin. The results are presented as the immunoreactive bands and the respective quantification. At 48 hpi., the BCL-2 level remained unaltered by either JEV infection or calpeptin treatment (Fig. 3A and B). In contrast, the proteolytic cleavage of BAX was markedly altered in JEV-infected cells. The results demonstrated that p18 BAX was significantly upregulated in JEV-infected cells compared to uninfected control cells, in which p18 BAX was undetectable (*p* < 0.0001, Fig. 3C). While the level of full-length p21 BAX in the infected cells seems to remain as found in uninfected control cells (Fig. 3D). Calpeptin treatment caused a notable and significant reduction in p18 BAX levels in JEV-infected cells (*p* < 0.0001, Fig. 3C). No effect of calpeptin alone on BAX cleavage was observed. The markedly declining levels of cleaved p18 BAX were observed to correlate with the reduction in calpain and caspase-3 activity (Fig. 2E and G) and the percentage of cell death (Fig. 2B) during JEV infection in SH-SY5Y cells. Furthermore, calpeptin treatment significantly reduced JEV-induced PARP cleavage, a hallmark of caspase-mediated apoptosis, compared to untreated infected cells (*p* < 0.05; Fig. 3E and F). These findings indicate that calpain proteolytic activity is responsible for p18 BAX generation in JEV-infected neuronal cells, and that inhibition of calpain-mediated BAX cleavage attenuates JEV-induced apoptosis.

### Inhibition of calpain suppressed p18 BAX and Cyt *c* mitochondrial accumulation

**Fig. 4:**
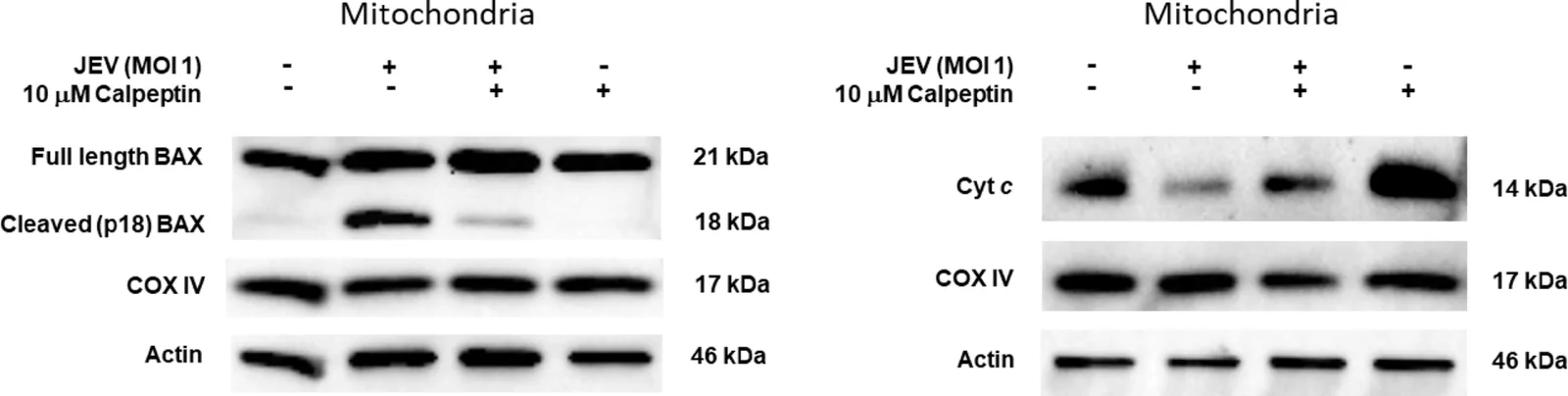
The effect of calpeptin on BAX and cytochrome *c* (Cyt *c*) accumulation in JEV-infected SH-SY5Y cells. Cells were pretreated with 10 µM calpeptin for 1 h prior to JEV infection at an MOI of 1, maintained in standard medium for 24 h, and then treated with 10 µM calpeptin for a further 24 h before harvesting at 48 hpi. The infected cells were collected, and mitochondrial proteins were extracted by mitochondria fractionation. The immunoreactive detection of BAX (full length BAX and p18 BAX), and Cyt *c* was performed by western blotting. COX IV was used as a marker of mitochondrial fraction and actin was used as a marker of cytosolic fraction and a loading control. The immunoblots shown are from one representative experiment. The replicate blots are presented in Supplementary Fig. 8

To further investigate whether calpain-mediated BAX cleavage contributes to mitochondrial dysfunction during JEV infection, we examined the expression of p18 BAX and Cyt *c* in mitochondrial fraction of JEV-infected SH-SY5Y cells following calpain inhibition by calpeptin. Mitochondria fractionation was performed at 48 hpi. Consistent with our previous findings, JEV-infected cells at 48 hpi exhibited an increase in p18 BAX levels in the mitochondrial fraction and a corresponding significant decrease in Cyt *c* levels in the mitochondrial fraction, compared with uninfected control cells (Fig. 4), which represents the release of Cyt *c* into the cytosol. Treatment with calpeptin significantly reduced mitochondrial p18 BAX accumulation and correspondingly attenuated the decrease in mitochondrial Cyt *c* levels in JEV-infected cells (Fig. 4), indicating that calpain activity is required for both p18 BAX mitochondrial targeting and subsequent Cyt *c* release. Taken together, these findings support that the JEV-induced neuronal death proceeds through a calpain-dependent intrinsic apoptotic pathway, in which calpain-mediated BAX cleavage to p18 fragment, which promotes mitochondrial permeabilization and subsequent Cyt *c* release.

### Calpeptin treatment does not alter the JEV replication

**Fig. 5:**
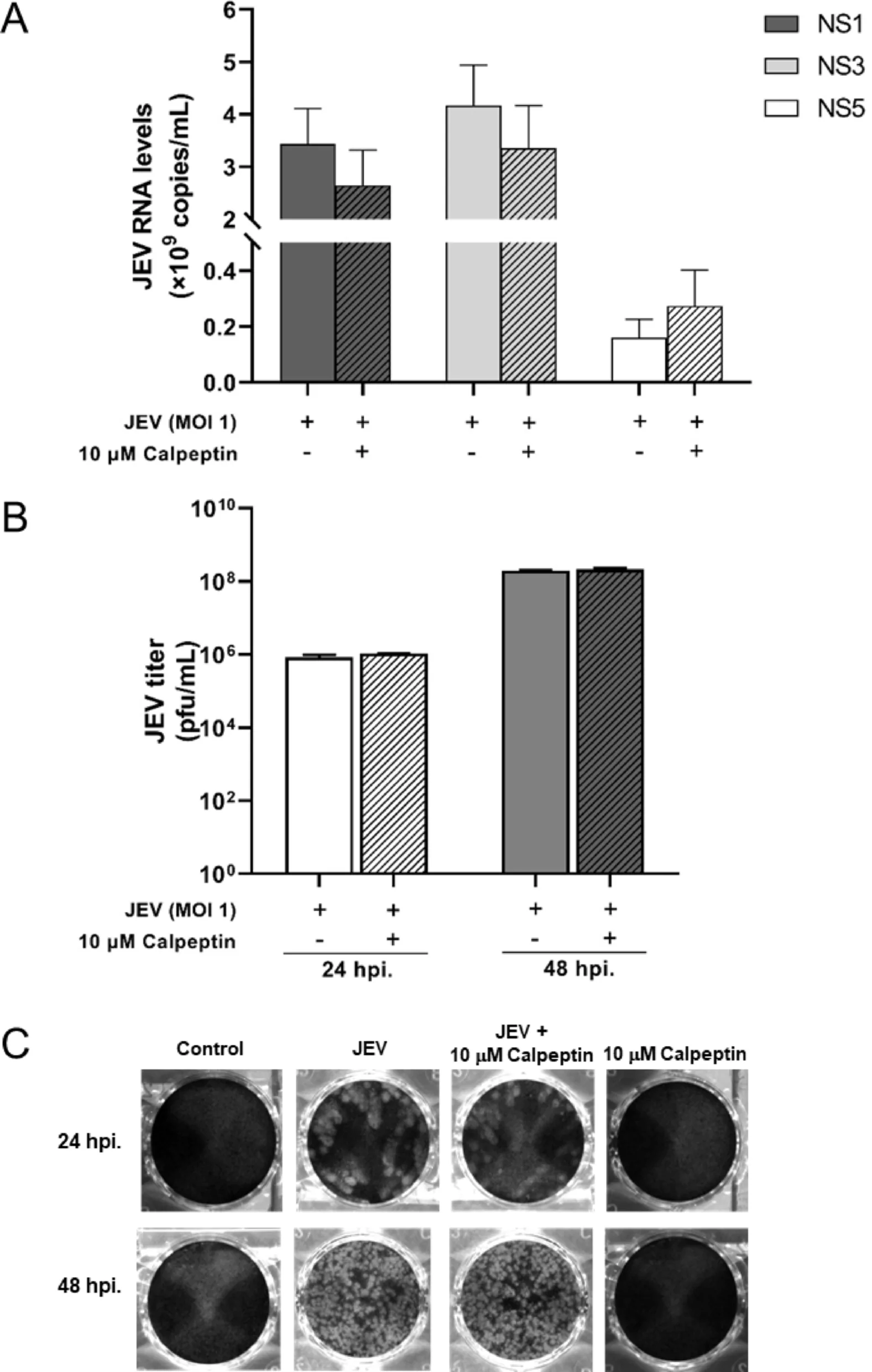
The effect of calpeptin treatment on JEV replication. Cells were pre-treated with 10 µM calpeptin and infected with 1 MOI of JEV and then maintained in calpeptin for the final 24 h prior to collecting the culture medium at 48 h. The experimental culture supernatants were collected to quantify JEV titer by dPCR and plaque assay (**A**) Absolute quantification at 48 hpi of JEV-RNA levels in copies/mL (NS1, NS3, and NS5 genes) was measured by dPCR. (**B**) The viral titers are expressed in pfu/mL determined by plaque assay. (**C**) The representative plaque formation assay results were shown. Images for the control group were obtained from undiluted culture supernatant, whereas images of JEV-infected group are shown at a 10^− 5^ dilution. Data represent means ± SD of three independent experiments. One-way ANOVA and Tukey–Kramer multiple comparison tests were performed, and no statistical significance was detected

Following the treatment of JEV-infected cells with calpeptin, the inhibitory effect on viral replication was evaluated by dPCR and plaque assay at the 48 hpi. The absolute quantification of JEV genomic RNA in experimental culture supernatant at 48 hpi was quantified using specific probes and primers targeting nonstructural NS1, NS3 and NS5 genes. As shown in Fig. 5A, JEV genomic RNA indicating new viral progeny reached to 3.43 ± 0.67, 4.17 ± 0.77, and 0.16 ± 0.07 × 10^9^ copies/mL detected by NS1, NS3 and NS5, respective. The JEV-infected cells treated with 10 µM of calpeptin slightly decreased the viral NS1 and NS3 RNA levels whereas viral NS5 RNA level was slightly increased when compared with JEV-infected cell control without calpeptin treatment. However, no significant differences in all studied genes were observed. To confirm the negatively influence of calpeptin on JEV replication. The JEV viral titers at 24 hpi and 48 hpi were examined by plaque assay. As shown in Fig. 5B, without calpeptin treatment, the titer of JEV reached to 8.36 ± 1.27 × 10^5^ and 1.95 ± 0.06 × 10^8^ pfu/mL at 24 hpi and 48 hpi, respectively. After calpeptin treatment, JEV still efficiently replicated which titer were 10.06 ± 0.21 × 10^5^ and 2.09 ± 0.21 × 10^8^ pfu/mL at 24 hpi and 48 hpi, respectively. There were no significant differences observed on JEV titer between with or without calpeptin treatment. Taken together, our results suggest that calpeptin treatment has no effect on the JEV viral replication.

## Discussion

We previously demonstrated that JEV elevates the formation of an N-terminal truncated version of BAX (p18 BAX), triggering mitochondria-mediated apoptosis [10]. This study investigates the underlying mechanism of p18 BAX generation during JEV infection in SH-SY5Y cells. Using western blotting and subcellular fractionation, and cell viability assays, we reveal for the first time that JEV infection promotes neuronal apoptosis via the activation of calpain, as evidenced by increased levels of the cleavage fragment of spectrin (145 kDa SBDP). This activation was accompanied by elevated generation of the pro-apoptotic p18 BAX fragment. Elevation of p18 BAX levels were associated with Cyt *c* release, caspase-3 activation, increased cleaved PARP, and ultimately apoptotic cell death in JEV-infected cells. This observation is supported by our previous report showing that p18 BAX translocate to mitochondria, resulting in the release of Cyt *c* and inducing the activation of the caspase cascade at the late stage of JEV infection [10]. Importantly, calpain inhibition with calpeptin significantly reduced p18 BAX formation in the late stage of JEV infection and attenuated JEV-induced neuronal death. Our findings demonstrate a crucial role for the calpain-mediated BAX cleavage pathway in JEV-induced neuropathogenesis and suggest calpain inhibition as a potential therapeutic strategy.

**Fig. 6:**
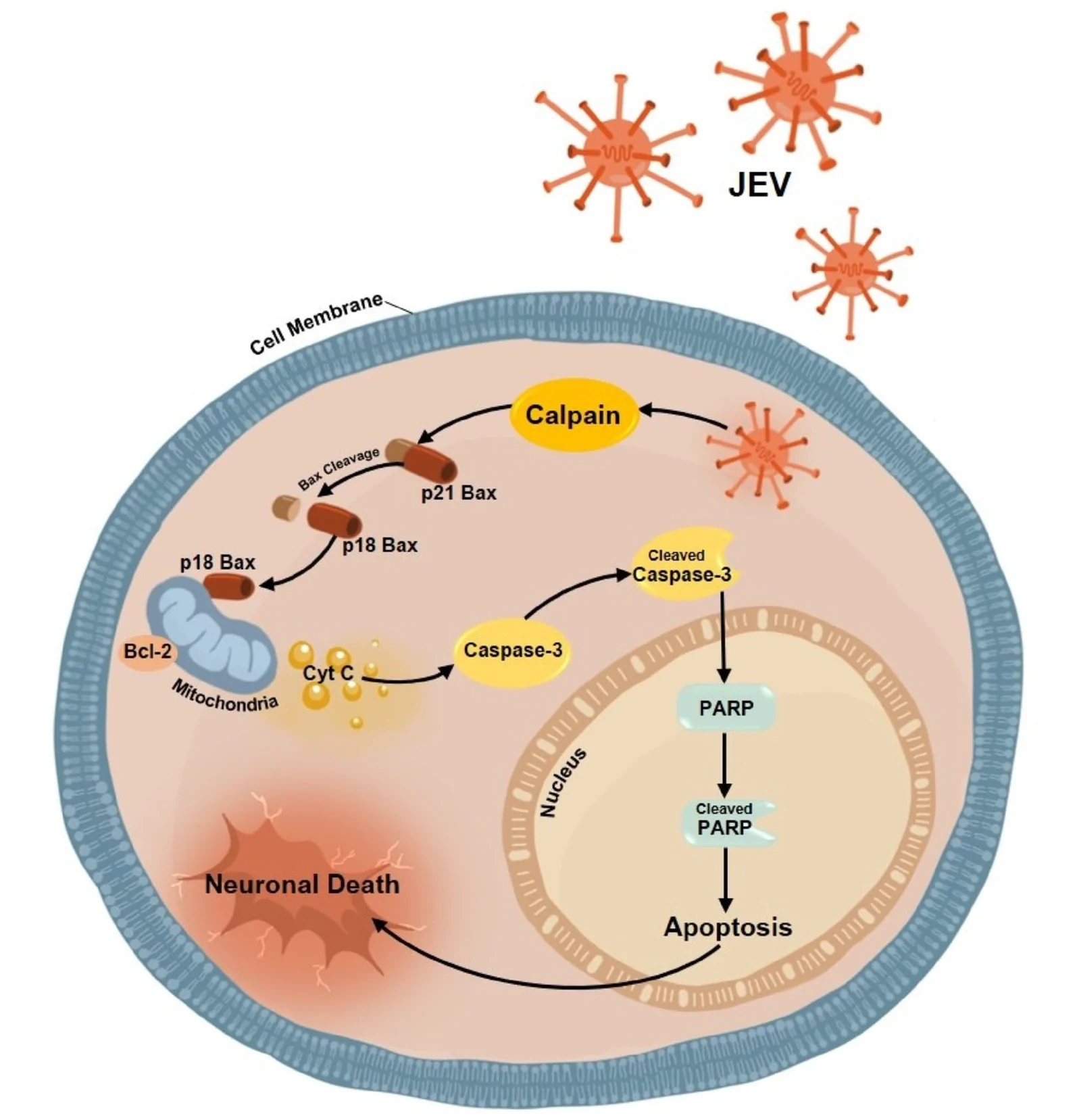
Schematic illustration of the proposed Japanese encephalitis virus (JEV)-induced neuronal death through calpain-mediated BAX cleavage to initiate the mitochondrial apoptosis pathway. In the late stage of infection, JEV activates calpain which induces the process of cleavage of full-length BAX (p21) to generate the pro-apoptotic p18 BAX fragment. Cleaved p18 BAX translocates to mitochondria and effectively induces cytochrome *c* (Cyt *c*) release into the cytosol, leading to activation of the caspase cascade. Active cleaved caspase-3, in turn, cleaves cellular substrates such as PARP (poly ADP-ribose polymerase 1). The cleavage of PARP initiates apoptosis, which ultimately leads to the death of neuronal cells. (Image created with PowerPoint.)

The identification of calpain as a key player in JEV-induced neuronal apoptosis aligns with previous studies on other neurotropic RNA viruses. Calpain activation has been implicated in the pathogenesis of IAV [24], human immunodeficiency type virus 1 (HIV-1) [20], EV71 [22], echovirus 1 (EV1) [21], CVB3 [19], Theiler’s murine encephalomyelitis virus (TMEV) [32] and reovirus [33] infections. Our study is the first to demonstrate this mechanism in JEV infection, extending the relevance of calpain-mediated apoptosis to flavivirus neuropathogenesis. Calpains are a family of calcium-dependent proteases that play a crucial role in the pathogenesis of viral infections. Previous reports have shown that calpains can exhibit both pro-viral and anti-viral effects, depending on the specific virus and host cell type involved. Viral infection triggers calcium mobilization that contributes to the activation of calpains. In some virus infections, calpain activity contributes to cleaving viral proteins, which is essential for their replication and spread [24, 28, 34]. On the other hand, virus infection activates calpain by altering calcium flow to induce the host cell-death mechanism by triggering a caspase-independent apoptotic pathway [22, 28, 35]. We showed here that the activation of calpain by JEV infection can subsequently induce cleavage of p21 BAX to generate p18 BAX, which appears to be a critical step in JEV-induced neuronal apoptosis. This N-terminally truncated p18 BAX protein is known to be more potent in inducing apoptosis than its full-length form [36–39]. As shown in vesicular stomatitis virus (VSV) infection, the infected cells display decreased levels of anti-apoptotic proteins, including BCL-2 or BCL-xL, and increased levels of p21 BAX and p18 BAX which induces mitochondria-initiated caspase activation pathway [40, 41]. Our observation shows that calpeptin treatment significantly reduced p18 BAX levels and decreased apoptosis markers in both cleaved caspase-3 and PARP in SH-SY5Y-infected cells, suggesting that the calpain proteolysis is a potential mechanism of neuronal death in JEV infection. Furthermore, direct visualization of p18 BAX and Cyt *c* mitochondrial redistribution by subcellular fractionation following calpain inhibition provides additional confirmation of the intrinsic mitochondrial apoptotic pathway (Fig. 4). The calpain inhibition experiments thus demonstrate calpain-specific effects on both upstream protease activity of calpain, direct BAX cleavage (p18 BAX generation) and then release of Cyt *c* to cytosol, downstream caspase-3 activation, and terminal apoptotic execution (PARP cleavage) during viral infection. This sequence supports calpain as one of the upstream protease initiating JEV-induced neuronal apoptosis (Fig. 6). Therefore, we propose that during JEV infection, calpain is activated and cleaves the pro-apoptotic BAX protein, thereby generating p18 BAX. This cleaved form demonstrates enhanced pro-apoptotic activity by homodimerizing and translocating to mitochondrial membranes, thereby promoting Cyt *c* release [10, 36, 37, 42, 43]. The released cytosolic Cyt *c* then triggers the intrinsic apoptotic cascade by activating caspase-3, leading to its processing and enzymatic activation, which ultimately induces neuronal apoptotic cell death [10, 11, 37, 43].

The p18 BAX is unable to form heterodimers with antiapoptotic BCL-2 family members that are capable of overcoming the inhibition of apoptosis and thus potently accelerate apoptosis by disrupting mitochondrial integrity [14, 42]. We observed no significant changes in BCL-2 levels following JEV infection or calpeptin treatment. This finding is consistent with our previous studies in SH-SY5Y cells, in which BCL-2 expression remained stable during high MOI JEV Beijing-1 infection and did not effectively suppress virus-induced cell death during late infection stages (48–72 hpi.) [10, 11]. These observations suggest that the pro-apoptotic shift in JEV-infected neurons is primarily driven by BAX activation rather than BCL-2 downregulation. The variability in BCL-2 regulation reported across different JEV studies reflects the complexity of viral-host interactions and several important experimental variables. Different viral strains, infection doses, experimental timepoints, and cell type-specific responses significantly influence apoptotic pathways activation, which also contribute to the varying in BCL-2 expression levels [13, 44–46]. Our findings demonstrate that calpain-mediated generation of the highly pro-apoptotic p18 BAX provides a potent pro-apoptotic signal that drives neuronal death independently of BCL-2 regulation, especially in the late stage of JEV infection, representing a possible mechanism in JEV neuropathogenesis.

Calpain activation exerts a significant influence on the outcomes of viral infections. Consequently, there is a growing interest in exploring calpains as potential targets for antiviral therapeutic interventions. A previous study demonstrated that using a calpain inhibitor reduced the production of EV1 particles after the microinjection of EV1 RNA into the cells, and they effectively inhibited the synthesis of viral RNA in the host cells [21]. Inhibition of calpain inhibits endocytosis of SARS-CoV to the host cell and inhibits the progeny virus replication [27, 47]. Furthermore, treatment with a calpain inhibitor has been demonstrated to impede the deleterious effects of viral infection. Treatment with a calpain inhibitor can interact with the calcium-binding site and inhibit reovirus-induced apoptosis in L929 cells [48]. Inhibiting calpain by both calpain inhibitor and calpastatin overexpression attenuated apoptosis in CVB3-infected mice by decreasing inflammation and suppressing endoplasmic reticulum stress-associated protein levels and apoptotic-related proteins [26]. Inhibition of calpain activity in reovirus strain 8B infection in neonatal mice prevented myocardial injury as well as decreased the serum creatine phosphokinase levels leading to weight gain among animals treated with a calpain inhibitor [33]. The inhibition of calpain by using protease inhibitor significantly reduced calpain activity in the hippocampus of TEMV-infected mice, which protected neurons from death, preserved cognitive performance, and suppressed seizure escalation when therapy was initiated 36 h after the disease onset of TEMV infection however, it was not affect the viral biology [32]. Calpain inhibitors treatment leads to a reduction of inflammation, and platelet activation during SARS-CoV-2 infection [28]. These reports demonstrate that the effects of calpains can vary depending on the virus, cell type, and stage of infection. The protective effect of calpain inhibition, as observed in our study, suggests the potential for calpain inhibitors to reduce virus-induced neuronal cell death, which serves as a potential therapeutic strategy for JEV encephalitis. Currently, there are no specific antiviral treatments for JEV infection, and management is largely supportive. Importantly, our results demonstrate that calpeptin exerts no effect on JEV replication, as confirmed by both dPCR and plaque assay (Fig. 5), indicating that its protective effect acts exclusively on the host apoptotic pathway rather than on viral biology. This distinction is therapeutically relevant, as it suggests that calpain inhibition could potentially mitigate neuronal damage without imposing selective pressure on the virus. The present findings suggest that targeting calpain could potentially mitigate neuronal damage and improve outcomes in JEV encephalitis.

The present study demonstrates the calpain-mediated BAX cleavage pathway in JEV-induced neuronal apoptosis; however, several limitations should be acknowledged. First, all experiments were conducted at the single timepoint of 48 hpi, selected based on the peak of p18 BAX accumulation established in our previous work. A comprehensive time-course analysis would be necessary to characterize the temporal dynamics of calpain activation and BAX cleavage during the full course of JEV infection and to determine whether calpain inhibition offers sustained neuroprotection beyond this period. Next, our study employed calpeptin, a broad-spectrum inhibitor of both calpain-1 and calpain-2, which does not allow identification of the specific isoform responsible for BAX cleavage in this context. Furthermore, calpeptin exhibits known off-target inhibitory activity against other cysteine proteases, including cathepsins, which cannot be entirely excluded as contributing factors in the observed effects. The isoform-specific investigations could identify whether calpain-1 or calpain-2 predominates in BAX cleavage, enabling precision therapeutic targeting. Our experiments were performed exclusively in SH-SY5Y neuroblastoma cells infected with a single viral strain (JEV Beijing-1) at a single MOI. Since SH-SY5Y cells are an undifferentiated neuroblastoma line that does not fully recapitulate the physiology of mature neurons, extending these findings to primary neuronal cultures, differentiated SH-SY5Y cells, other JEV-permissive neuronal cell types, other JEV genotypes, as well as to JEV-infected animal models would strengthen biological relevance and translational applicability. Finally, future studies incorporating genetic approaches such as BAX knockout or siRNA knockdown studies would reveal the critical role of BAX and calpain in the neuronal death pathway induced by JEV infection and may reveal additional regulatory mechanisms. These investigations would advance therapeutic development for viral encephalitis.

## Conclusions

In conclusion, this study clarified the role of the calpain-mediated BAX cleavage mechanism in JEV-induced neuronal apoptosis (Fig. 6). This finding not only advances our understanding of JEV neuropathogenesis but also identifies calpain as a potential therapeutic target. Modulating the calpain-BAX pathway could be a potential strategy for antiviral therapies. Future studies exploring the role of calpain in JEV replication and investigating direct interactions between viral proteins and calpain or its regulators could unveil novel aspects of JEV pathogenesis, which would enhance the biological relevance of viral encephalitis.

## Supplementary Information

Supplementary Material 1

## Data Availability

The datasets used and/or analysed during the current study are available from the corresponding author on reasonable request.
