# Supplementary material for: Inhibition of calpain-mediated BAX cleavage attenuates Japanese encephalitis virus-induced apoptosis in SH-SY5Y neuroblastoma cells
Source: Virol J. 2026 Jul 2;23:220. doi: 10.1186/s12985-026-03242-x (PMC13599172; doi:10.1186/s12985-026-03242-x)
Supplement: Supplementary file 1 [file 12985_2026_3242_MOESM1_ESM.pdf]

Immunoblot for anti-αII-Spectrin: original exported image from Image Lab software "Export for Analysis" mode

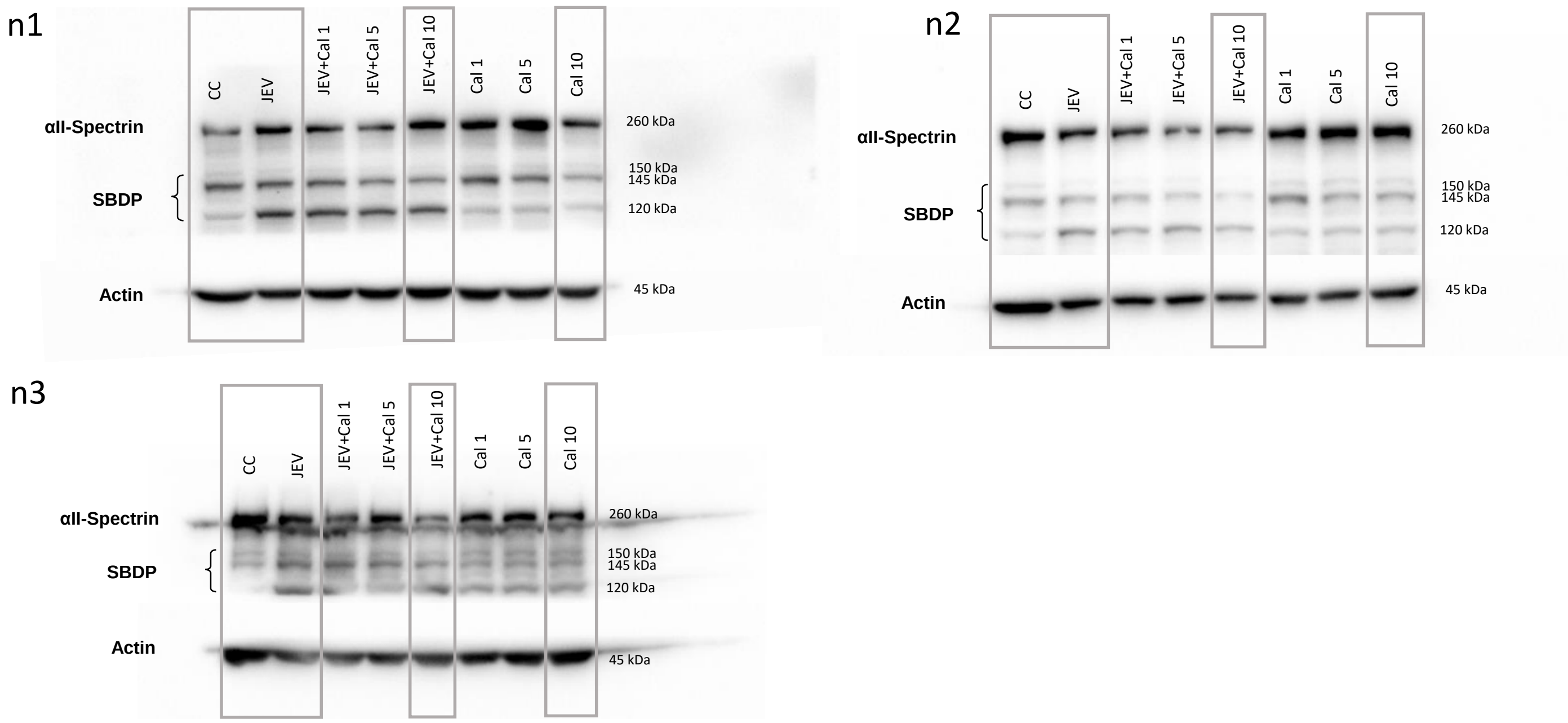

Supplementary Figure 1. Western blot analysis was performed by varying the concentration of calpeptin, and 10 μM calpeptin was selected for report. The bands that were selected for intensity analysis are displayed in the square frame. The n3 blot, which is the representative immunoreactive for αII-Spectrin (260 kDa), SBDP (145/150 kDa and 120 kDa), and Actin (45 kDa), is shown in Figure 2D.

Immunoblot for anti-caspase3: original exported image from Image Lab software "Export for Analysis" mode

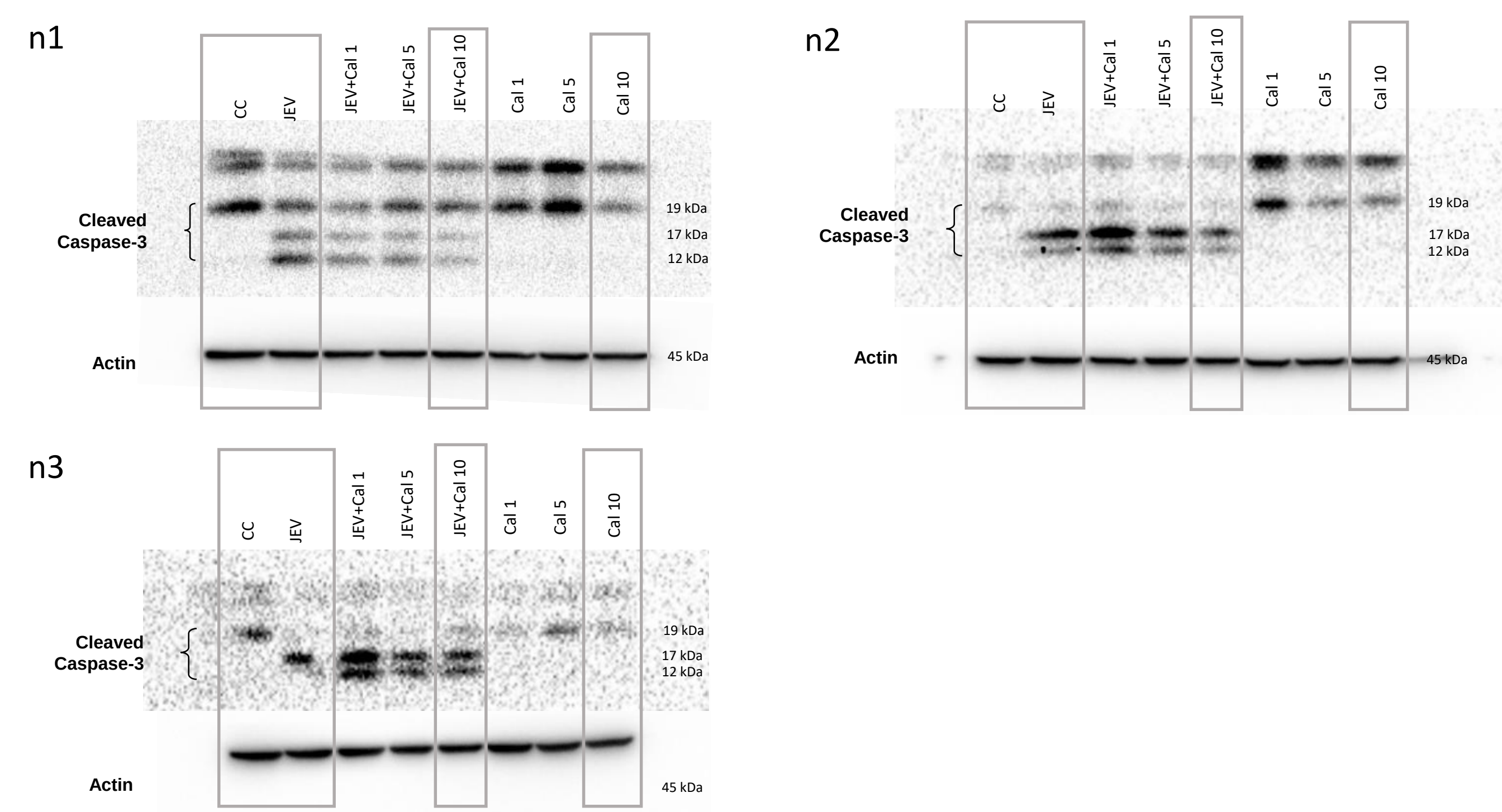

Supplementary Figure 2. Western blot analysis was performed by varying the concentration of calpeptin, and 10 μM calpeptin was selected for report. The bands that were selected for intensity analysis are displayed in the square frame. The n1 blot, which is the representative immunoreactive for cleaved caspase 3 (19, 17 and 12 kDa), and Actin (45 kDa), is shown in Figure 2F.

Immunoblot for anti-BCL-2: original blots

n1

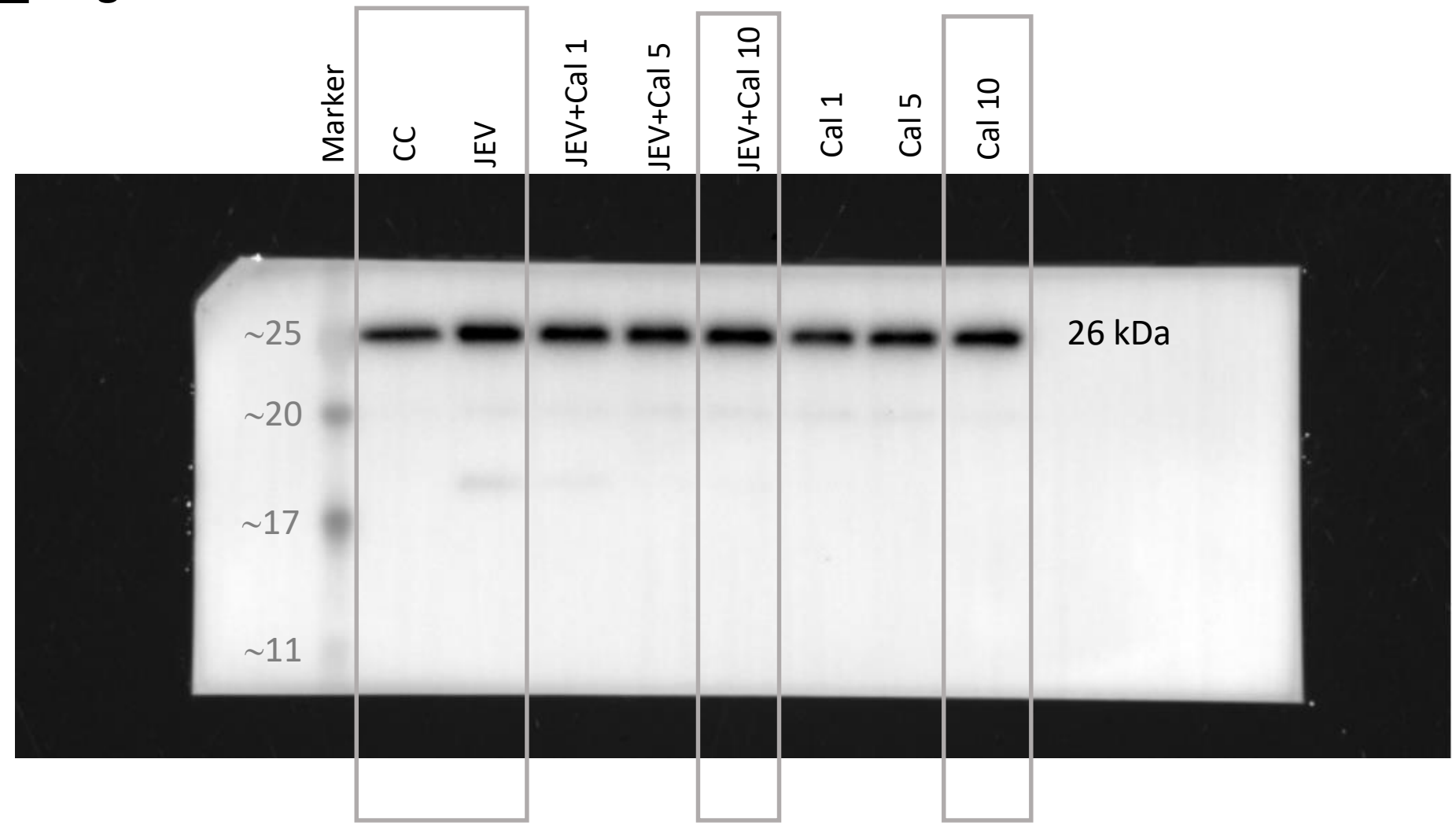

n2

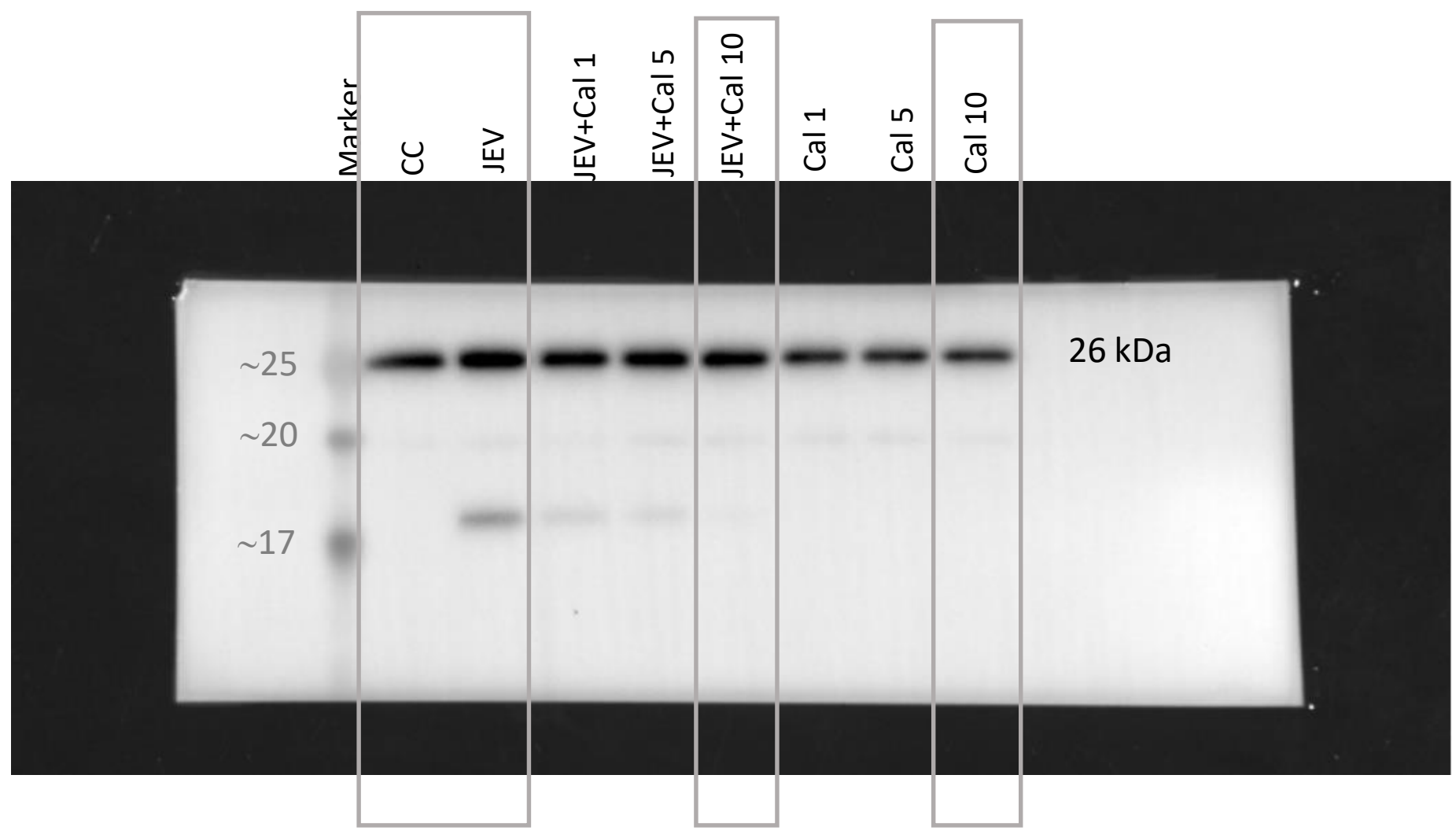

n3

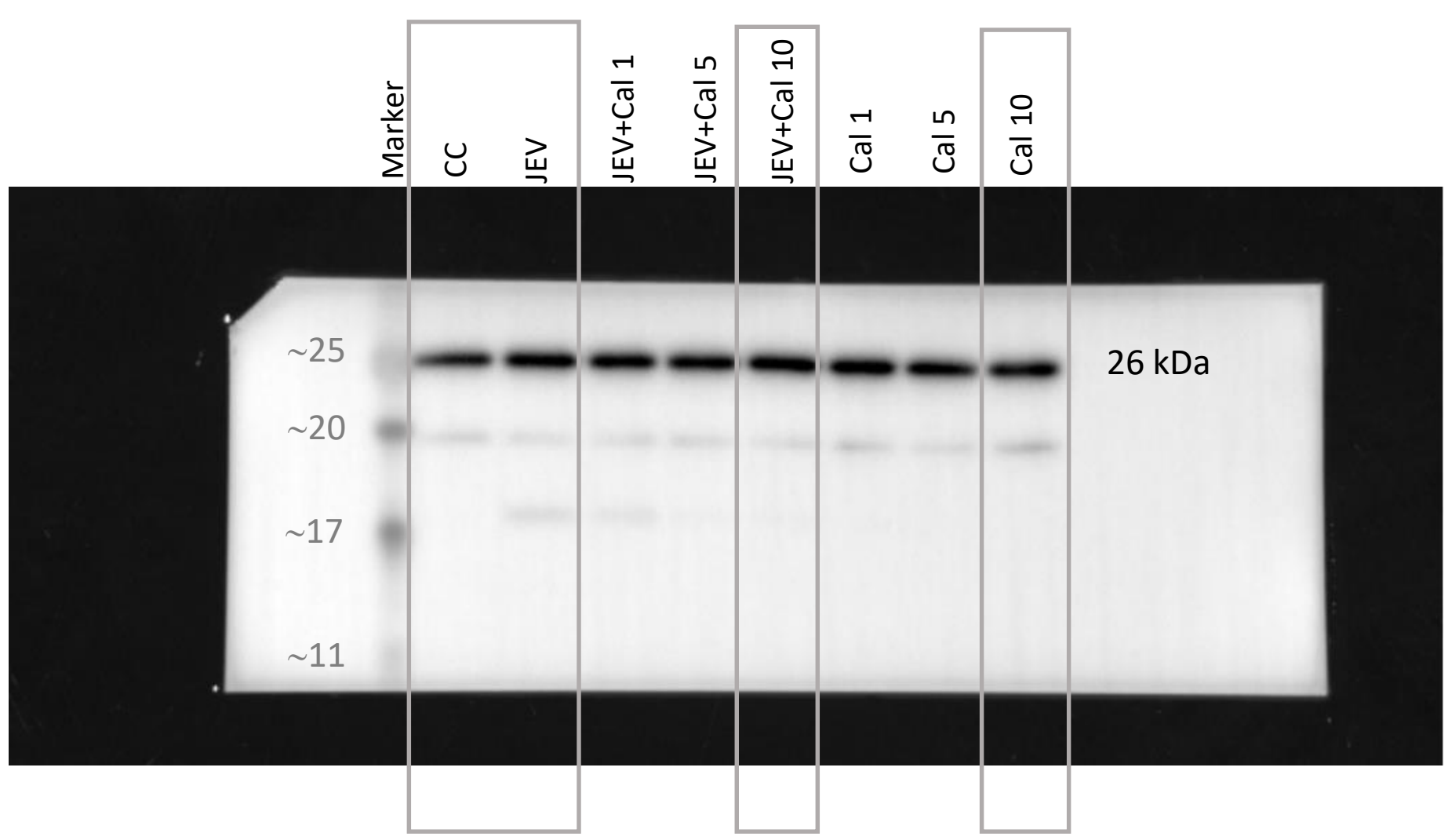

Supplementary Figure 3. Western blot analysis was performed by varying the concentration of calpeptin, and 10 μM calpeptin was selected for report. The bands that were selected for intensity analysis are displayed in the square frame. The n3 blot, which is the representative immunoreactive for BCL-2 (26 kDa), is shown in Figure 3A.

Immunoblot for anti-BAX: original blots

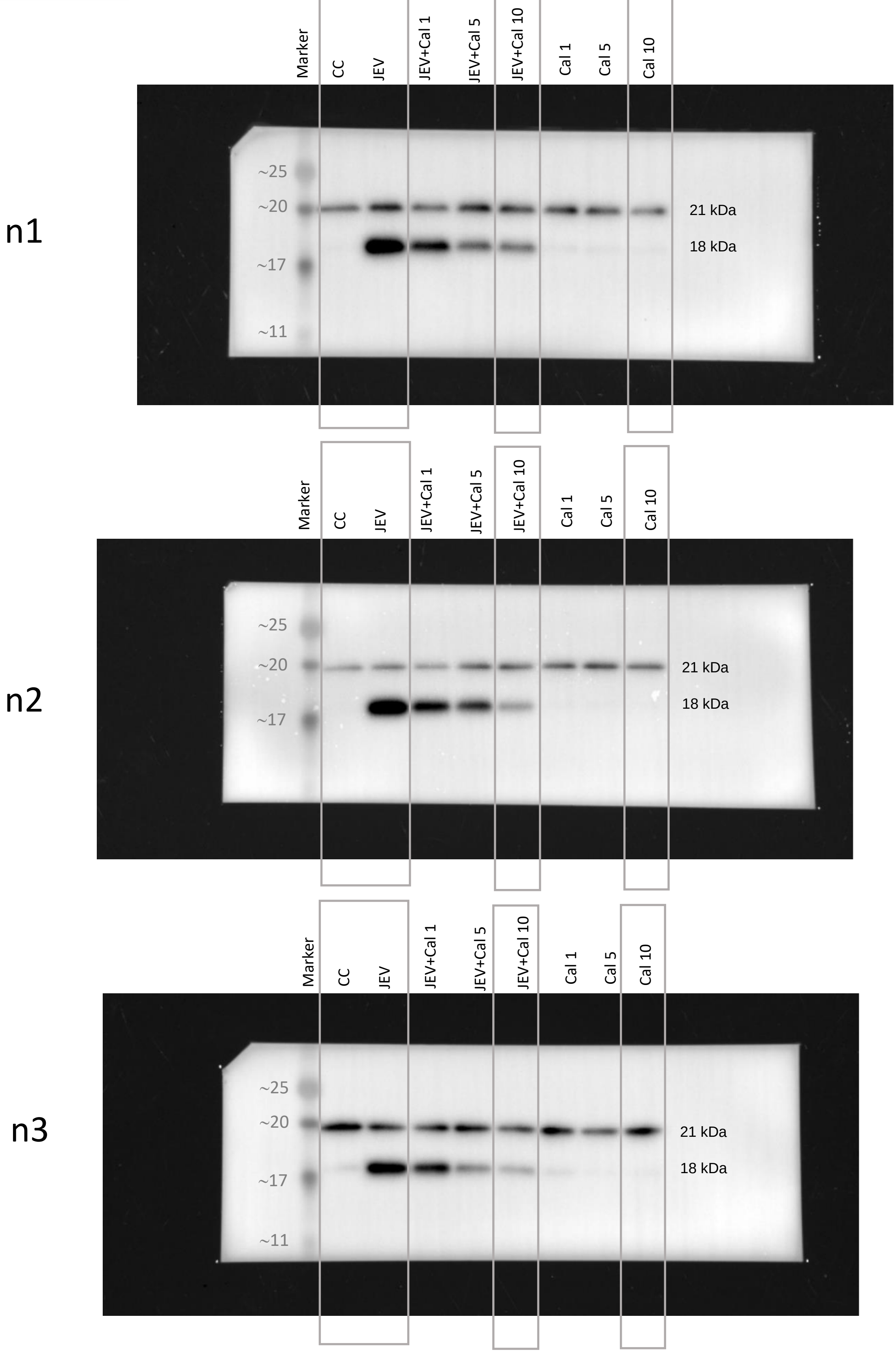

Supplementary Figure 4. Western blot analysis was performed by varying the concentration of calpeptin, which demonstrated a significant effect at 10 µM. The bands that were selected for intensity analysis are displayed in the square frame. The n2 blot, which is the representative immunoreactive for BAX (p21 and p18), is shown in Figure 3B.

Immunoblot for anti-Actin: original blots
(for BAX and BCL-2 normalization)

n1

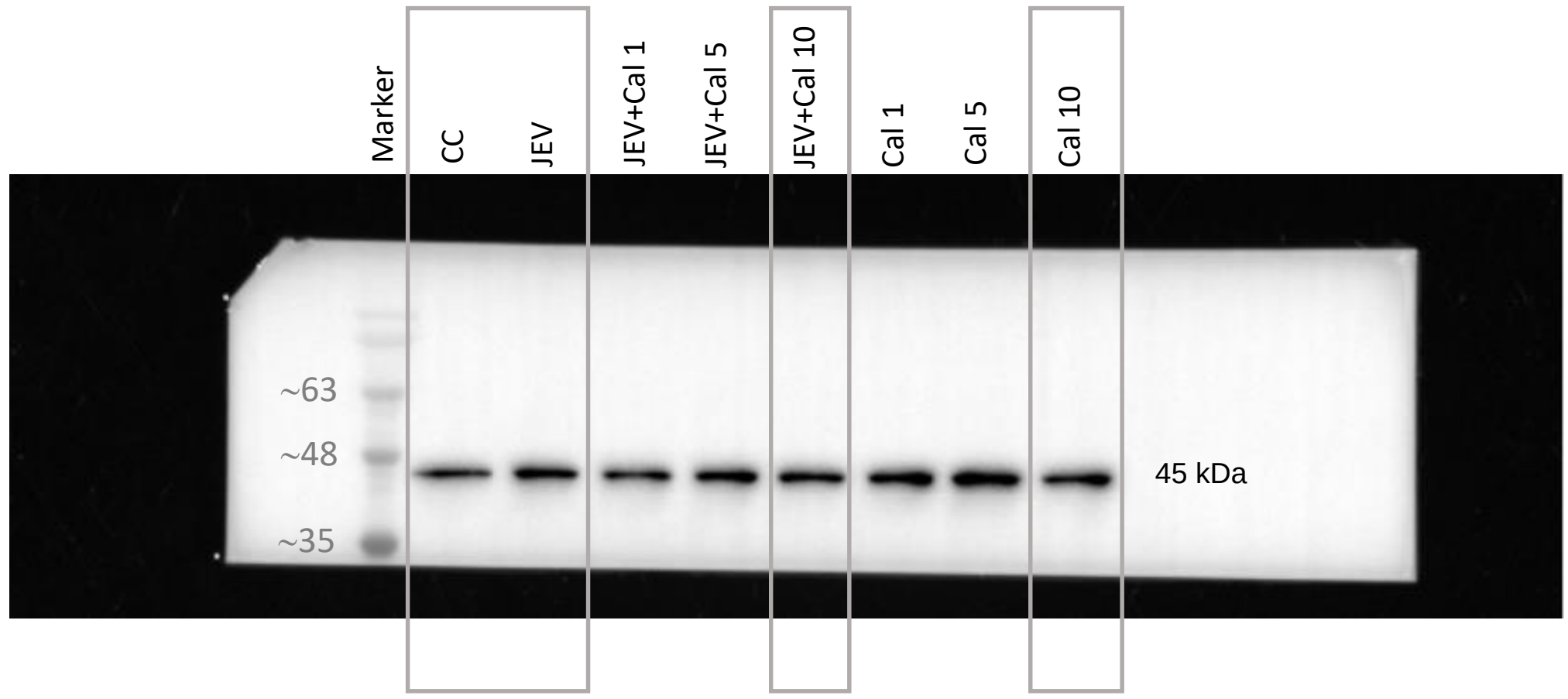

n2

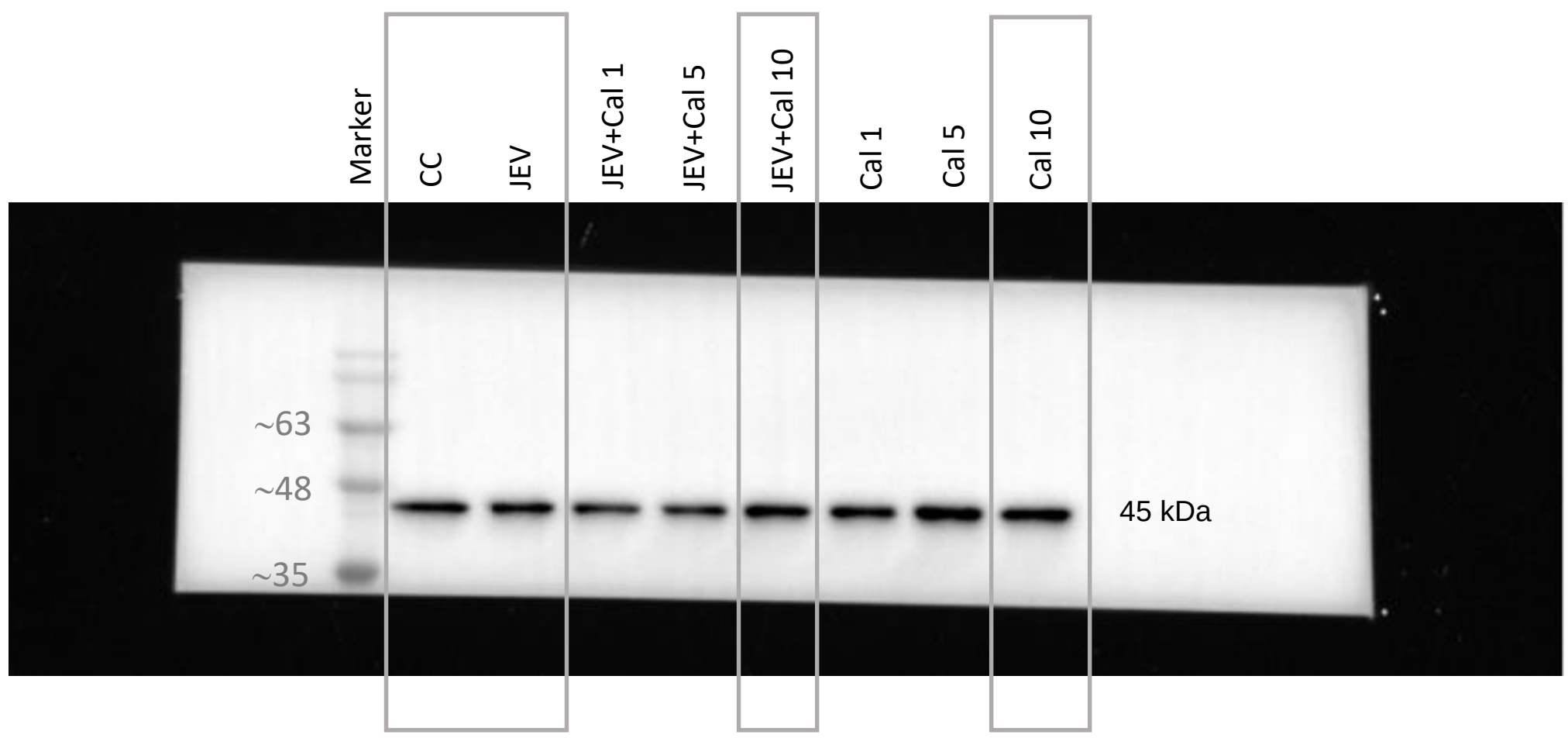

n3

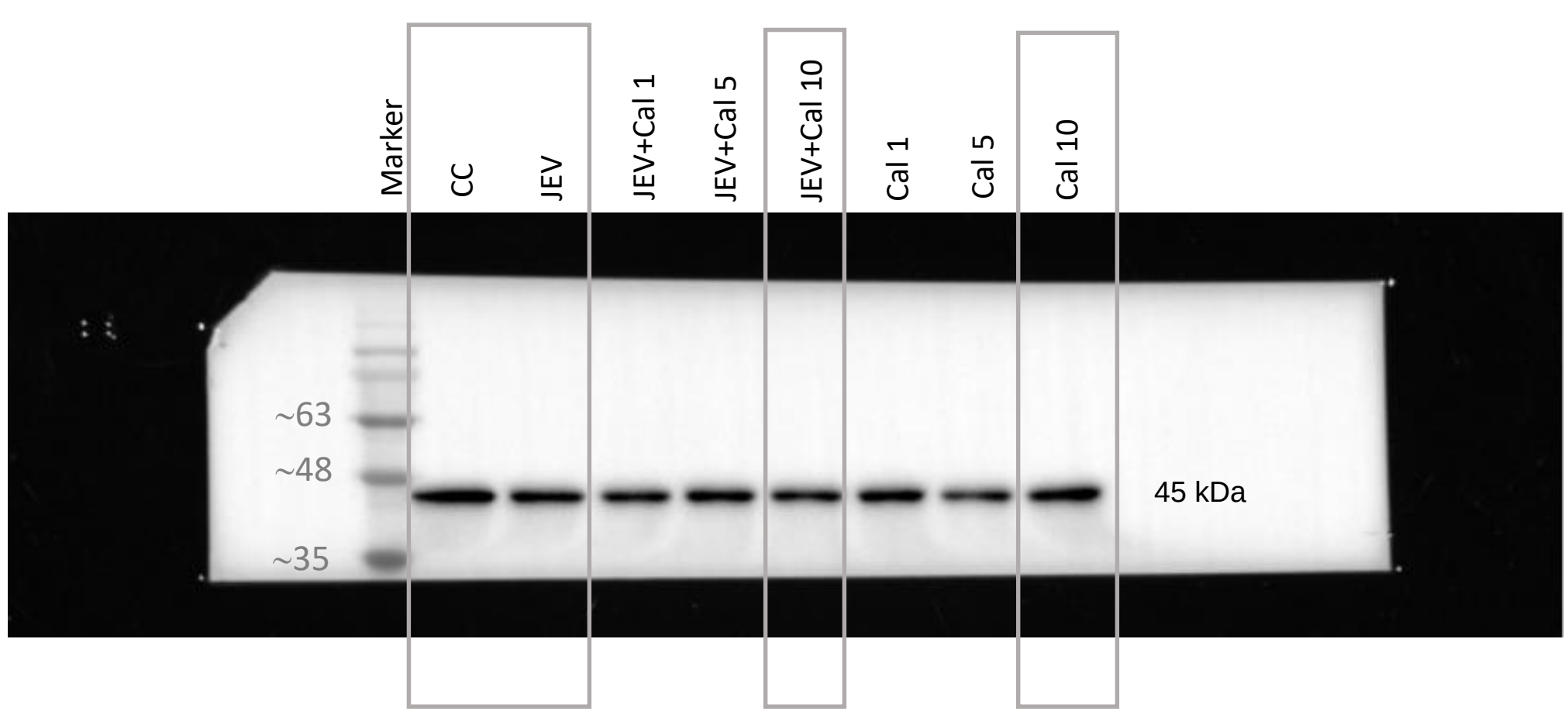

Supplementary Figure 5. Western blot analysis was performed by varying the concentration of calpeptin, and 10 μM calpeptin was selected for report. The bands that were selected for intensity analysis are displayed in the square frame. The n2 and n3 blot of Actin, which are the representative immunoreactive of Actin (45 kDa), is shown in Figure 3 for BAX and BCL-2, respectively.

Immunoblot for anti-PARP: original blots

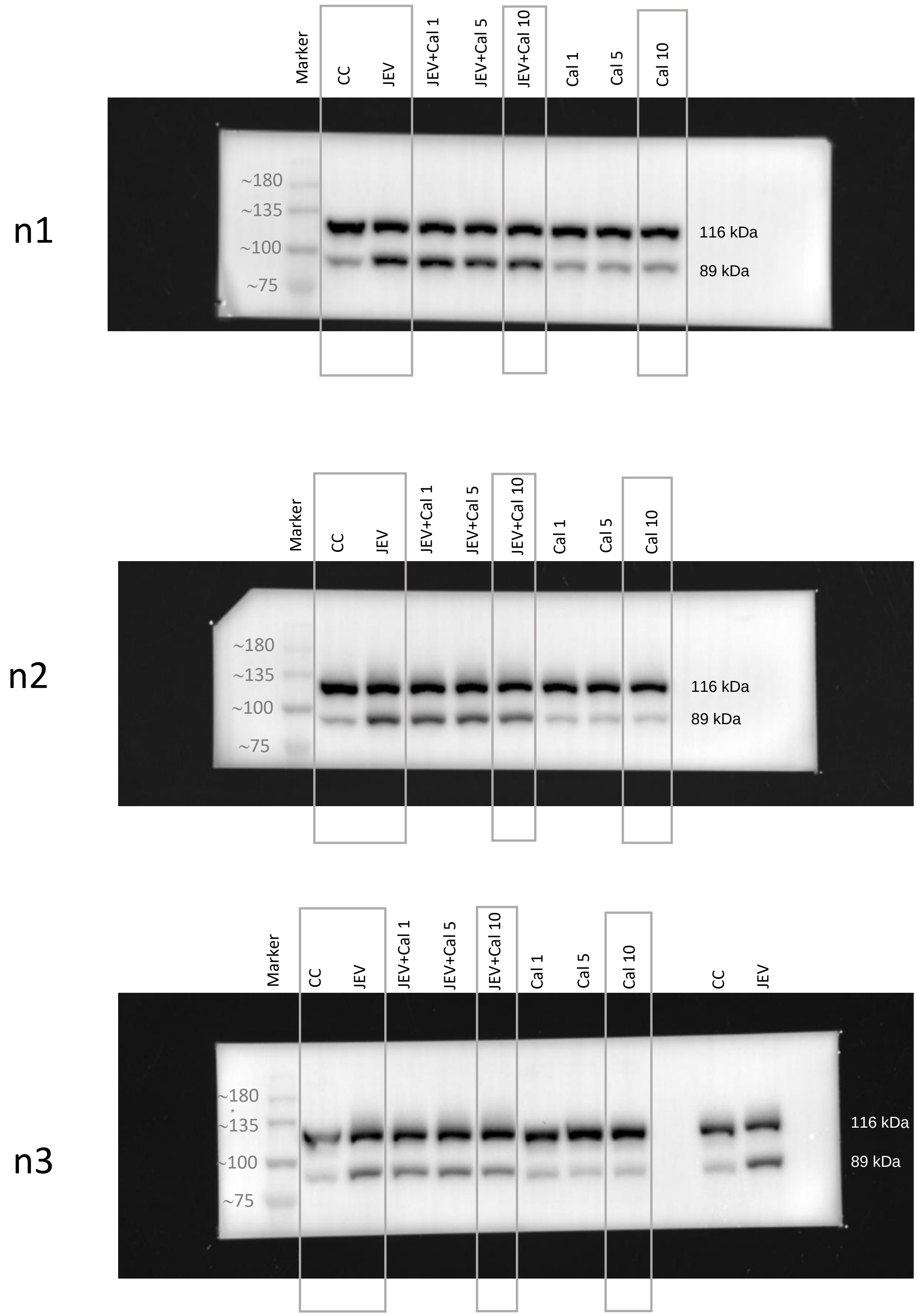

Supplementary Figure 6. Western blot analysis was performed by varying the concentration of calpeptin, which demonstrated a significant effect at 10 µM. The bands that were selected for intensity analysis are displayed in the square frame. The n1 blot, which is the representative immunoreacitve for PARP (116 kDa) and cleaved PARP (89 kDa), is shown in Figure 3C.

<u>Immunoblot for anti-Actin:</u> original blots
(for PARP normalization)

n1

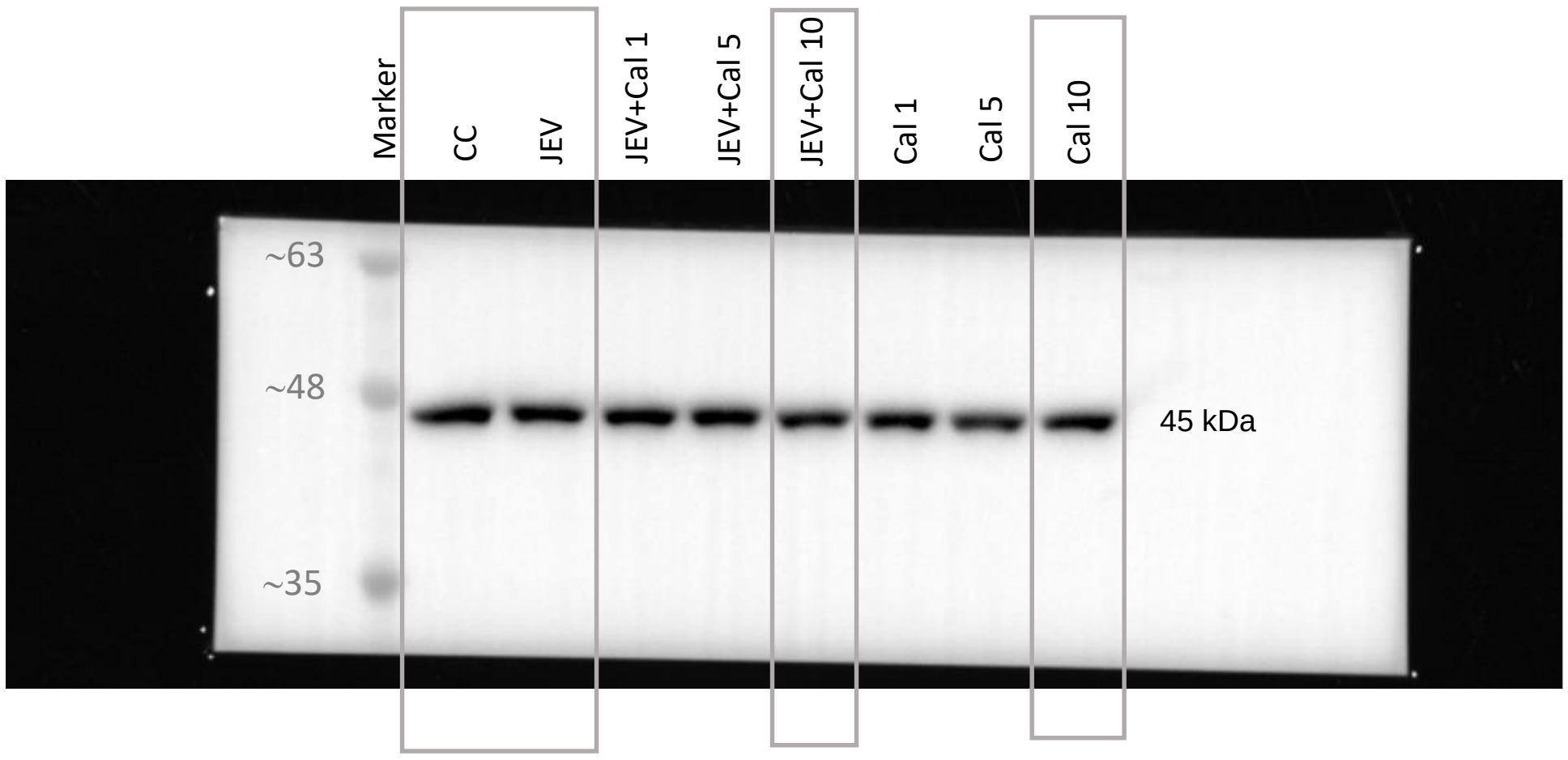

n2

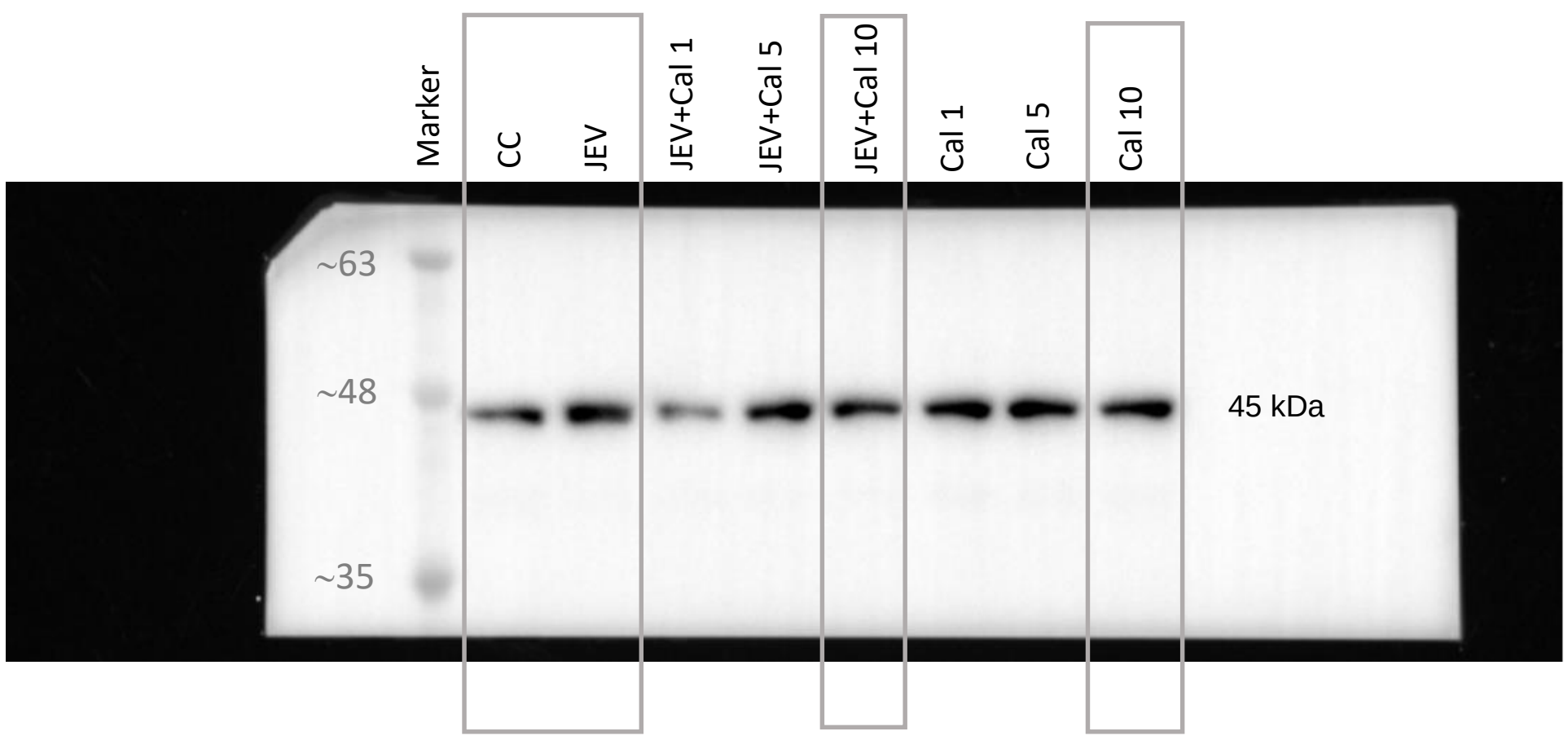

n3

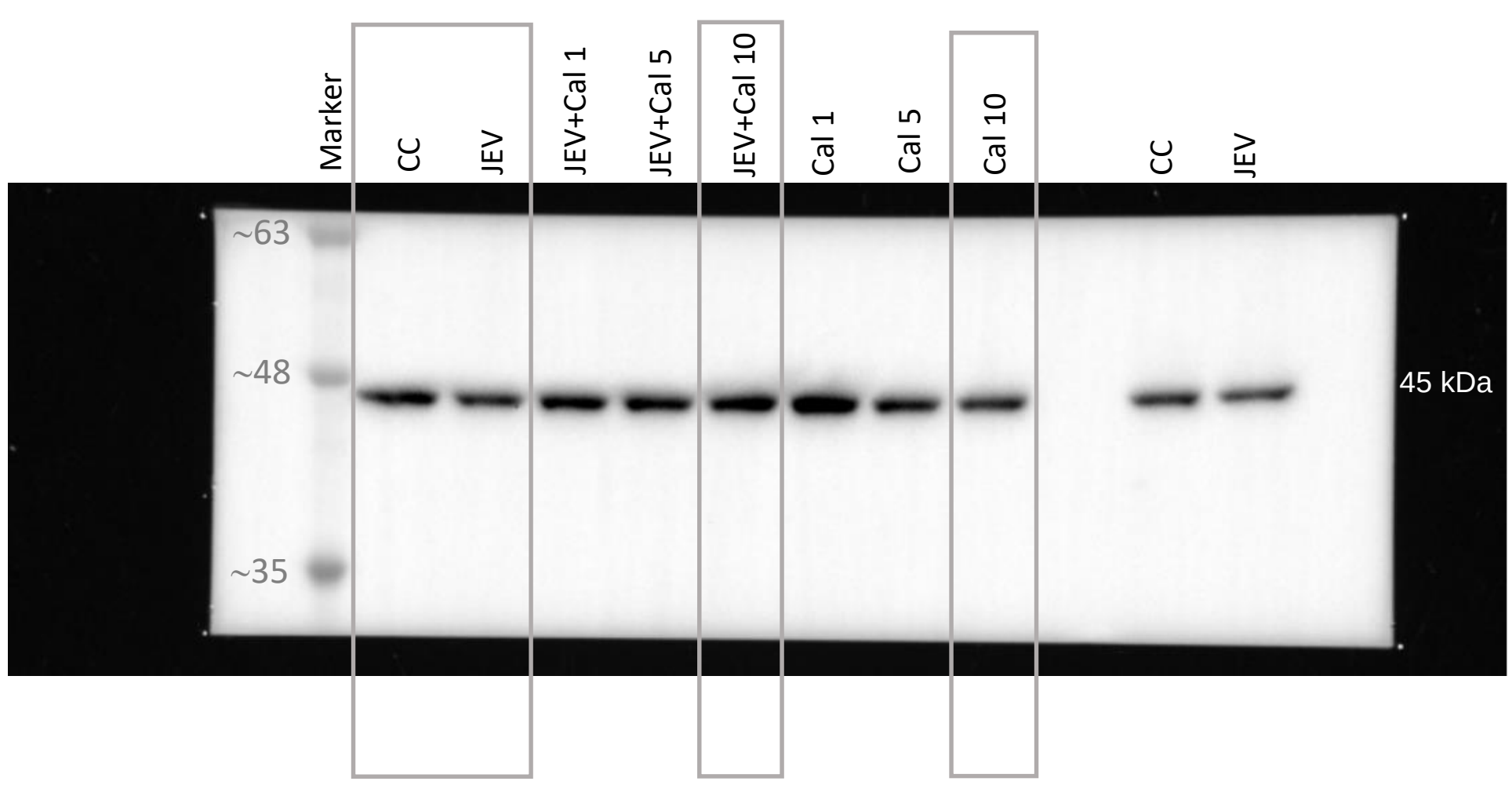

Supplementary Figure 7. Western blot analysis was performed by varying the concentration of calpeptin, and 10 µM calpeptin was selected for report. The bands that were selected for intensity analysis are displayed in the square frame. The n1 blot, which are the representative immunoreactive for Actin (45 kDa), is shown in Figure 3C.

Mitochondria fraction
Immunoblot for anti-BAX: original replicate blots

n1

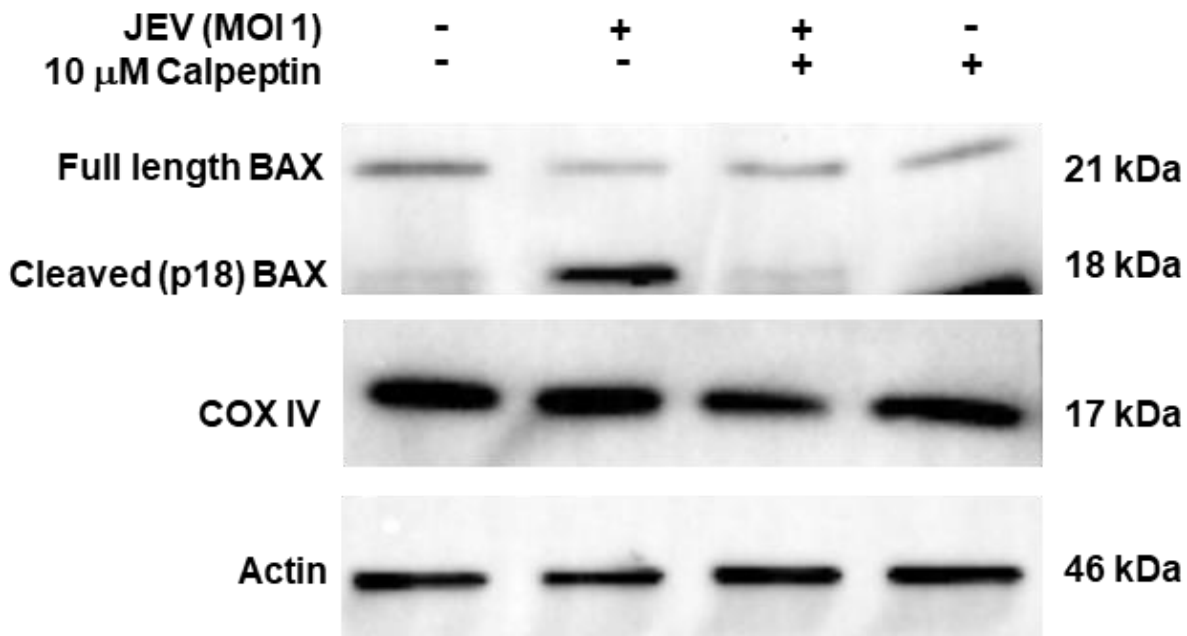

n2

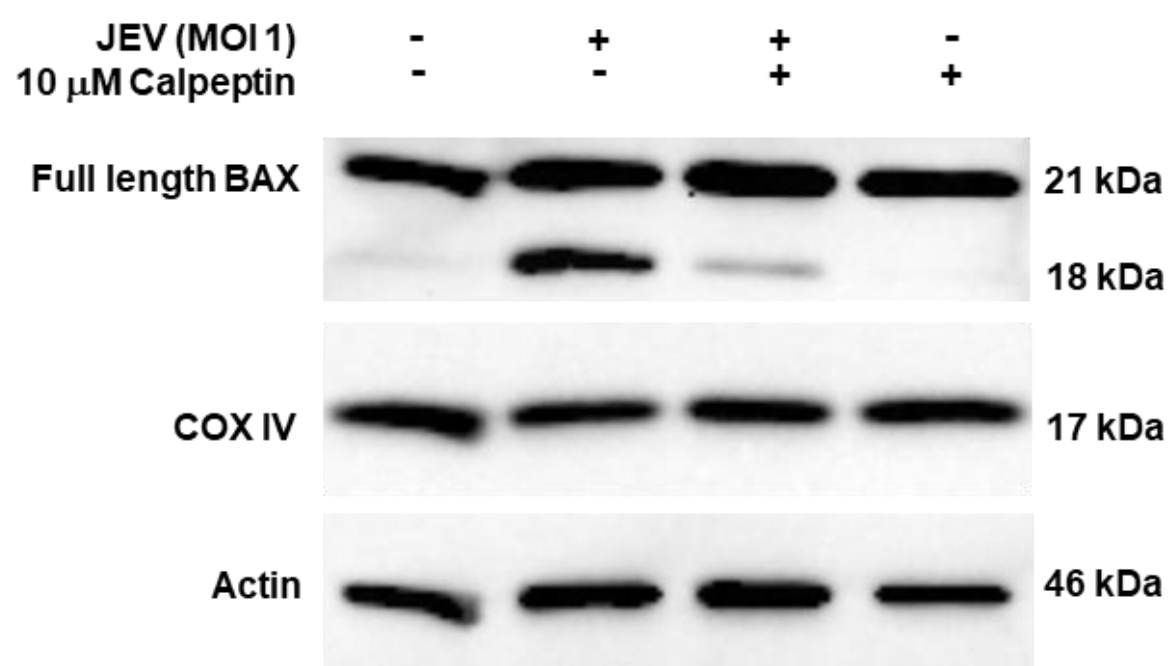

n3

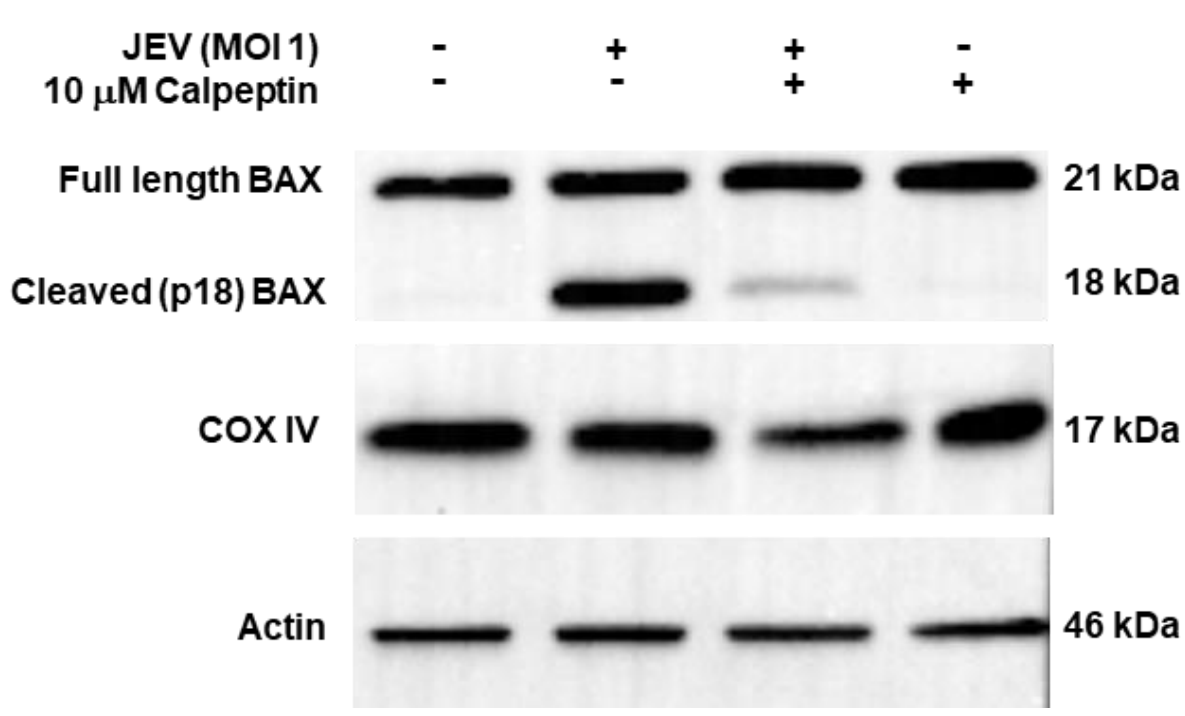

Mitochondria fraction
Immunoblot for anti-Cyt c: original replicate blots

n1

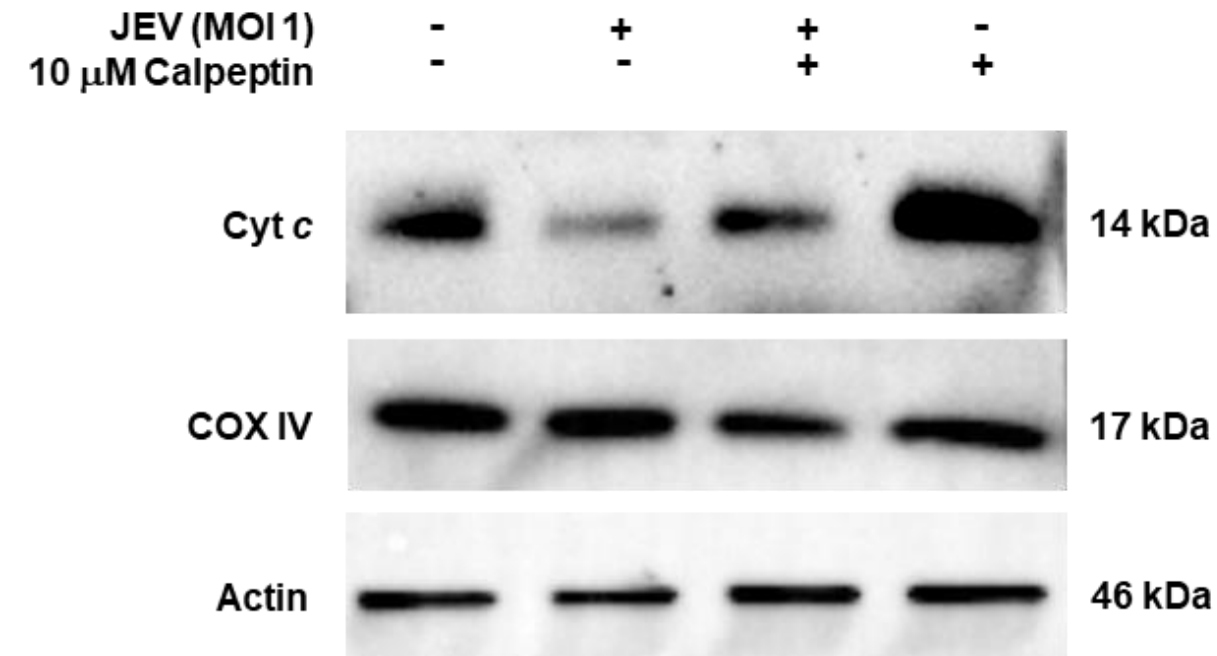

n2

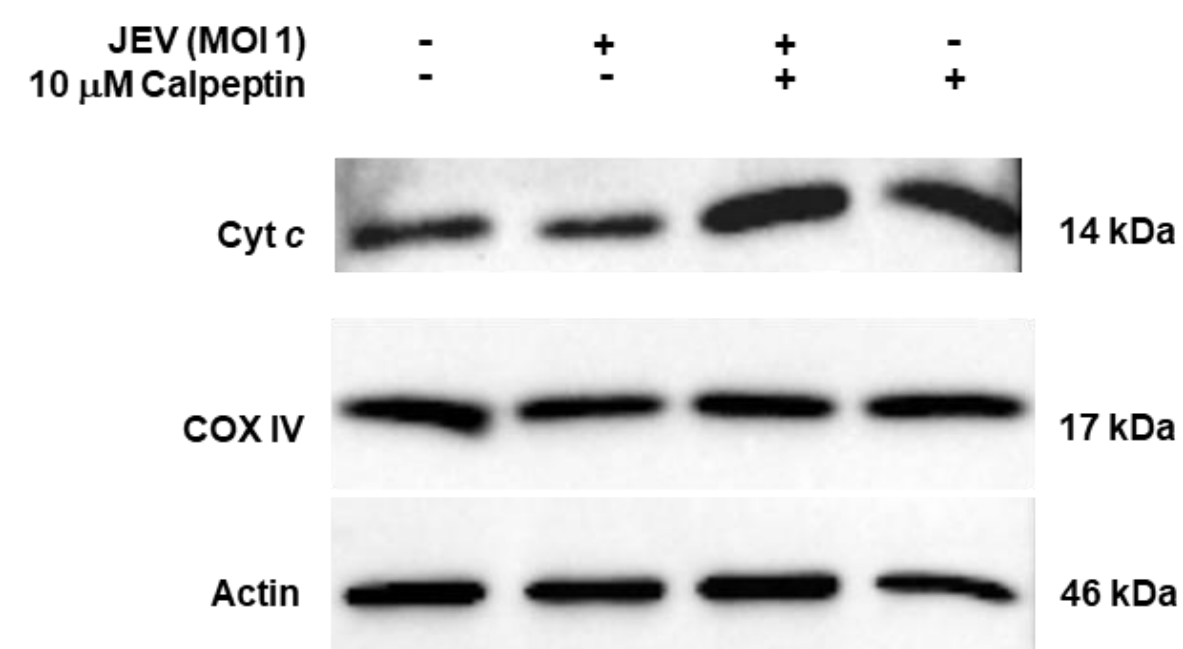

Supplementary Figure 8. Western blot was performed for detection *left panel*: BAX (full length BAX and p18 BAX), and *right panel*: cytochrome *c* (Cyt *c*) in mitochondria fractions. The n2 and n1 blots, which are the representative immunoreactive for BAX (21 and 18 kDa) and for Cyt c (14 kDa), are shown in Figure 4. COX IV (17 kDa) was used as a marker of mitochondrial fraction and actin (46 kDa) was used as a marker of cytosolic fraction
